# Interaction Between Familial Transmission and a Constitutively Active Immune System Shapes Gut Microbiota in *Drosophila melanogaster*

**DOI:** 10.1534/genetics.116.190215

**Published:** 2017-04-14

**Authors:** Rupal Mistry, Ilias Kounatidis, Petros Ligoxygakis

**Affiliations:** Laboratory of Cell Biology, Development and Genetics, Department of Biochemistry, University of Oxford, OX1 3QU, United Kingdom

**Keywords:** *Drosophila*, constant inflammation, gut microbiota, innate immunity, Genetics of Immunity

## Abstract

Resident gut bacteria are constantly influencing the immune system, yet the role of the immune system in shaping microbiota composition during an organism’s life span has remained unclear. Experiments in mice have been inconclusive due to differences in husbandry schemes that led to conflicting results. We used *Drosophila* as a genetically tractable system with a simpler gut bacterial population structure streamlined genetic backgrounds and established cross schemes to address this issue. We found that, depending on their genetic background, young flies had microbiota of different diversities that converged with age to the same Acetobacteraceae-dominated pattern in healthy flies. This pattern was accelerated in immune-compromised flies with higher bacterial load and gut cell death. Nevertheless, immune-compromised flies resembled their genetic background, indicating that familial transmission was the main force regulating gut microbiota. In contrast, flies with a constitutively active immune system had microbiota readily distinguishable from their genetic background with the introduction and establishment of previously undetectable bacterial families. This indicated the influence of immunity over familial transmission. Moreover, hyperactive immunity and increased enterocyte death resulted in the highest bacterial load observed starting from early adulthood. Cohousing experiments showed that the microenvironment also played an important role in the structure of the microbiota where flies with constitutive immunity defined the gut microbiota of their cohabitants. Our data show that, in *Drosophila*, constitutively active immunity shapes the structure and density of gut microbiota.

MICROBIAL populations inhabit the mucosal surfaces of animals and contribute to the development of their immune system [reviewed in Hill and Artis (2010)]. This is particularly the case for intestinal bacteria that additionally enhance resistance to infection by pathogenic bacteria (Vollaard and Clasener 1994). The composition and structure of the commensal bacterial populations inhabiting the organism’s epithelia depend extensively on exogenous factors, the earliest and most important of which is maternal transmission. It has been shown that microbiota of littermate mice are more similar to each other than the microbiota of genetically identical mice from different mothers (Ley *et al.* 2005), while in humans the microbiota of siblings is more similar than that of unrelated individuals (Turnbaugh *et al.* 2009a). Diet is another environmental factor, which especially influences the composition of intestinal microbiota, as has been shown in germ-free mice colonized by human microbiota and switched from a low-fat to a high-fat/high-sugar diet (Turnbaugh *et al.* 2009b). In flies, when different *Drosophila* species feeding in the wild on sources as varied as flower, fruit, or mushroom were transferred to the same food, all individuals obtained the same microbiota (Chandler *et al.* 2011). In addition, these authors transferred isogenic flies with identical microbiota from the same food medium to different media and found that dominant bacterial species varied in relation to the food (Chandler *et al.* 2011).

In contrast to the above, results on the role of the immune system in shaping the composition of intestinal microbiota has been somewhat conflicting. We know that the presence of immunoglobulin A (IgA) promotes intestinal health, its secretion into the intestine representing a key mechanism for regulating commensal microbial communities; for review see Brandtzaeg (2013). Nevertheless, data concerning the impact of Toll-Like Receptor (TLR) signaling has been contradictory. For example, the cecal microbiota of TLR-5-deficient mice differed from wild-type littermate controls in over a 100 bacterial species (Vijay-Kumar *et al.* 2010). Moreover, MyD88-deficient mice harbored a cecal microbiota with higher levels of Rikenellaceae and Porphyromonadaceae (Wen *et al.* 2008), while MyD88 signaling in T cells was found to direct IgA-mediated control of microbiota (Kubinak *et al.* 2015). However, other studies found that mice deficient in MyD88 or in TLR2, TLR4, TLR5, and TLR9 were not detectably different in their intestinal microbiota from their wild-type littermates (Ubeda *et al.* 2012), pointing toward problems in the parental lineage and mice husbandry in previous papers. Recently, a heterologous model where transgenic rats on the Lewis background expressing human HLA-B27 and β2-microglobulin was studied for effects on their gut microbiome (Lin *et al.* 2014). Overexpression of both was needed to alter microbiota composition and a consistent trend was documented with differences between wild-type rats and the transgenic animals. However, this trend was independent of gut inflammation and differences with wild-type rats could be due to different founder microbiomes. Again, the question of whether immune activity influenced microbiota remained largely unanswered.

In fruit flies, derepression of nuclear factor kappa-light-chain-enhancer of activated B cells [NF-κB (nuclear factor-kappa B)]-dependent mucosal immunity altered the gut microbiota and reduced life span (Lhocine *et al.* 2008; Ryu *et al.* 2008; Bonnay *et al.* 2013; Dantoft *et al.* 2013). Normal host life span was restored when flies were cultivated under germ-free conditions. However, these studies focused on a limited region of the midgut (Ryu *et al.* 2008), examined only the culture-dependent microbiota (Dantoft *et al.* 2013), or did not analyze the microbiota structure (Lhocine *et al.* 2008; Bonnay *et al.* 2013). Therefore, it is still an open question in both mice and flies whether a defective or deregulated immune system influences the composition of gut microbial populations across the adult life span.

Here, we have used two fruit fly strains in the same genetic background to address the role of immunity in shaping gut microbiota during adult life. *Drosophila* immunity is transcriptionally mediated by NF-κB [reviewed in Kounatidis and Ligoxygakis (2012)]. Two signaling pathways, namely Toll and Imd, regulate nuclear entry of this transcription factor. These pathways are activated by pattern recognition receptors through the sensing of peptidoglycan, the major structural component of the bacterial cell wall. In this context, Gram-positive bacteria strongly activate the Toll pathway, whereas Gram-negative bacteria and Gram-positive bacilli mainly trigger the Imd pathway (Kounatidis and Ligoxygakis 2012). To address the potential role of immunity in shaping bacterial populations in the gut, we used two fly strains. One strain (*dif-key*) lacked both Toll and Imd activity by being mutant in the NF-κB homolog *dif* (mediates Toll signaling) (Rutschmann *et al.* 2000a) and in *kenny* (*key*), the IκB-kinase-γ (Inhibitor of Nuclear Factor-kappa B) (IKKγ) subunit homolog, which transmits the Imd signal to the NF-κB homolog Relish (Rutschmann *et al.* 2000b). The second strain (*pirk;trbd*) had a constitutively active immunity since it was deficient in *pirk* (Lhocine *et al.* 2008) and *trabid* (Fernando *et al.* 2014), two negative regulators of Imd. Therefore, the *dif-key* strain was immune-deficient while *pirk;trbd* flies had a constitutively active immune system. Importantly, both the *dif-key* and *pirk;trbd* mutations were incorporated in the same yellow-white (*yw*) background, often used as a background for genetic screens and building of transgenic strains (Venken *et al.* 2011). In addition, as a wild-type reference, we chose one of the strains of the *Drosophila* Genetics Reference Panel (DGRP) (Mackay *et al.* 2012).

We applied next-generation sequencing (NGS) of whole-gut samples to determine the bacterial landscape of the intestine and ascertain the relative contributions of genetic background, familial transmission, immune status, and microenvironment. We complimented this analysis by quantifying bacterial load across the life span, as well as studying the physiological status of the gut (length, cell density, cell death, and pH). We found that flies with constitutively active immunity (*pirk;trbd*) had a variable microbiota structure across the life span that was distinctive from their genetic background used as a control. Experiments to remove familial transmission by producing germ-free embryos showed that, indeed, a constitutively active immune system played a major role in shaping gut microbiota. Moreover, the gut microbiota structure of immune-compromised flies resembled their genetic background in conventionally-reared flies (CR) but was markedly different when embryos were placed in normal food. This indicated that, in this instance, familial transmission was more important than immune status and pointed toward a self-regulating microbiota. Nevertheless, cohousing flies with constitutively active immunity in the same culture bottle as their genetic background did influence their respective microbiota structure, indicating an interaction between the microenvironment and immunity.

## Materials and Methods

### Experimental set-up

We employed high-throughput NGS using the ION Proton to determine the structure and relative abundance of bacterial species residing in the gut during the life span, exploring the potential influence of immune status. We oversaturated our sequencing reads to remove among-sample differences. Following quality filtering (see *Materials and Methods*), all rarefaction curves tended to saturation (Supplemental Material, Figure S1). This indicated that all operational taxonomic units (OTUs) were representative of the total bacterial community of each sample. Reads were from 40 guts of synchronized flies (pooled from 10 guts each of four parallel cultures) at 2, 7, 14, 20, and 40 days post eclosion (PE), except *pirk;trbd*, which had an LT_50_ of 22 days (20) and therefore day 20 was the last time point. Our experiments were performed in both CR flies and in conditionalized (CZ) lines. The latter were axenic flies reassociated with normal food immediately after the dechorionation of their eggs. CZ flies were used to exclude familial transmitted bacteria. Crowding conditions in all experiments described were identical with 30 flies per vial.

#### Biological repeats:

Due to observed variations in gut microbiota composition from experiment to experiment (24), we performed biological repeats of the 7 and 20 PE time points in CR and 2 and 14 PE time points in CZ flies for all strains (for CR see Figure S2 and for CZ see Figure S3). These biological repeats were performed 10 fly generations (∼120 days) after our initial experiments with the same protocol (*i.e.*, each time point was a pool of 40 guts from 10 guts each of four parallel cultures). These repeats confirmed initial observations. More specifically, Principal Coordinate Analysis (PCoA plots in Figure S2 and Figure S3) of UniFrac distances (Lozupone and Knight 2005) showed that initial experiments and biological repeats of the same genotype and time point were clustered together, having the smaller UniFrac distance evaluated against any other pairwise UniFrac distance comparisons.

#### Drosophila stocks, genetics, and husbandry:

Flies were maintained on cornmeal and molasses medium at 25° on a 12-hr light-dark cycle. *Yw^67c23^* (Bl#6599) was used in repeated backcrosses with *dif-key* (Rutschmann *et al.* 2002) and *pirk;trabid* (Fernando *et al.* 2014). These mutant strains were backcrossed to *yw* for eight generations to create *yw*; *dif-key* and *yw*; *pirk;trabid*. *DGRP-208* (Bl#25174) was used as an additional wild-type control. Flies were housed in vials/bottles containing fresh food and food was changed every 2 days. After backcrossing and when strains were established, we calibrated for any founder effects by starting culture of all strains used in experiments from a single cross (one female–one male). Therefore, all flies used in downstream experiments were descendants of that single cross.

#### Culture-dependent identification of gut microbiota:

A single fly was homogenized in LB (L3397, Sigma [Sigma Chemical], St. Louis, MO) or De Man, Rogosa, and Sharpe (MRS) broth (69966, Sigma) and serial dilutions were plated on both nutrient agar plates, LB (L3272, Sigma), and MRS (69964, Sigma). Plates were incubated at 25° for 3 days. Colonies representing each morphological type obtained on the two different media used were streaked for isolation on MRS plates and incubated in the same incubators for a further 2 days. Colony PCR was performed using 16S rRNA eubacterial primers 27F (5′-AGAGTTTGATCCTGGCTCAG-3′) and 1492R (5′-GGTTACCTTGTTACGACTT-3′). PCR products were run on a 1.2% gel and sent for sequencing with Source BioScience.

### Culture-independent identification of gut microbiota and subsequent analysis

#### Sample collection:

Forty isolated guts of CR samples were collected at the selected time points (days 2, 7, 14, 20, and 40) and DNA extracted using a QIAamp DNA Mini Kit (QIAGEN, Valencia, CA) according to the manufacturer’s protocol. These were 10 guts each pooled from four vials running in parallel. The same scheme was followed for all biological repeats as well as CZ samples (see below).

Preparation of CZ samples involved housing adults of the four strains (*DGRP-208*, *yw*, *dif-key*, and *pirk;trbd*) in cages separately to lay eggs on apple juice plates. Around 100 embryos were collected and washed in 50% bleach for 2 min followed by rinsing in 70% ethanol and sterile water for 2 min each. Embryos were transferred to vials containing conventional food using a sterile paintbrush under a laminar hood. Vials were placed at 25° in the same incubators as CR flies. Sterile water was added every 2 days. At the selected time points (days 2, 14, and 20) 40 guts were collected and DNA extracted using a QIAamp DNA Mini Kit (QIAGEN) according to the manufacturer’s protocol.

Cohoused flies were prepared by housing adults of the four strains (*DGRP-208*, *yw*, *dif-key*, and *pirk;trabd*) in cages separately to lay eggs on apple juice plates. Around 50 embryos of each strain were collected together, thus, either *yw* and *dif-key* or *yw* and *pirk;trbd* were collected together and washed in 50% bleach for 2 min followed by rinsing in 70% ethanol and sterile water for 2 min each. In parallel with the gut extraction, DNA from a pair of legs (per sample) was kept to distinguish the *dif-key* from *yw* samples. Embryos were transferred to vials containing conventional food using a sterile paintbrush under a laminar hood. Vials were placed at 25° in the same incubators as CR and CZ flies and sterile water was added every 2 days. At the selected time points (days 2 and 14), 40 guts were collected and DNA extracted using a QIAamp DNA Mini Kit (QIAGEN) according to the manufacturer’s protocol.

#### 16S amplicon library preparation:

50 ng/μl of DNA was amplified with Phusion Polymerase [New England Biolabs (NEB), Beverley, MA] using primers that amplify the V3 region of 16S rRNA region (V3F: 5′-CCAgACTCCTACGGGAGGCAG-3′ and V3R: 5′-CGTATTACCGCGGCTGCTG-3′). The product was run on a 1.4% agarose gel and the band was excised using a QIAquick Gel Extraction Kit (QIAGEN). Library preparation was followed using the NEBNext Fast DNA Library Prep Set for Ion Torrent (NEB) and Ion Xpress Barcode Adaptors (NEB) according to the manufacturer’s protocol. The amplicon libraries were purified using Agencourt AMPure XP DNA purification beads (Beckman, Fullerton, CA) to remove primer dimers and contaminants according to the manufacturer’s protocol. Samples were size selected on a 2% E-gel (Invitrogen, Carlsbad, CA) and quantified using quantitative real-time PCR (QPCR) using KAPA library quantification kits for the Ion Torrent platform with the primers IT A PCR (5′-CCATCTCATCCCTGCGTGTC-3′) and IT trP1 (F: 5′-CCACTACGCCTCCGCTTTCCTCTCTATG-3′) and against Ion Torrent DNA Standards (KK4812, KAPA Biosystems). CZ samples were reamplified for an extra six cycles according to the manufacturer’s protocol in the NEBNext Fast DNA Library Prep Set (NEB) and requantified prior to sequencing.

#### DNA sequencing:

Samples were loaded on to Ion 314 Chip v2 (multiplexing 16 samples per run) using the Ion Chef System and sequenced using Ion Proton system, (Thermo Fisher Scientific) according to manufacturer’s protocol.

### Statistics

#### Statistical analysis of 16S libraries pipeline:

Sequencing data were analyzed on the Ion Reporter software (Thermo Fisher Scientific) using a custom designed metagenomics workflow version 5 with V3 primers and referenced against curated microSEQ 16S reference library v2013.1. Primers were detected at the single end in each read. Reads <125 bp (after trimming primers) were excluded and the minimum alignment coverage was set at 90%. Only OTUs with at least three reads were retained, calculated to account for the technical error of the Ion Proton. The genus percentage identity was set at 97% and the species percentage identity at 99%. The difference in percentage between the top hit and the next hit was set at 0.8%. Graphs were designed in GraphPad Prism version 6.

#### Statistical analysis for sample comparisons:

**Figure 1:**
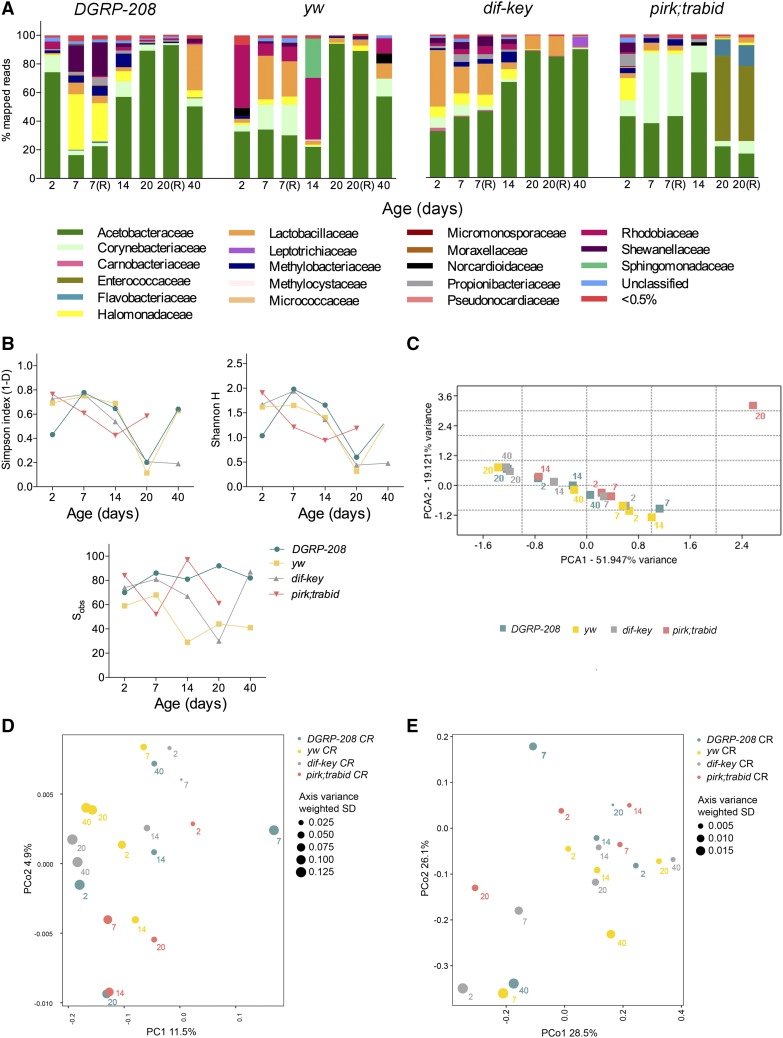
Microbiota of conventionally reared (CR) isolated guts. (A) The relative abundance of bacterial families detected in dissected guts from CR flies in two controls (*DGRP-208* and *yw*) and mutant strains (*dif-key* and *pirk*;*trbd*) across life span as revealed by 16S rRNA sequencing. (B) α diversity indices (Simpson’s and Shannon H indices) and total number of observed families (S_obs_) for all four strains across their life span. (C) Principal Component Analysis (PCA) of the bacterial community of the four strains in CR conditions. (D) Principal Coordinate Analysis (PCoA) of unweighted UniFrac distances. (E) PCoA of weighted UniFrac distances.

**Figure 2:**
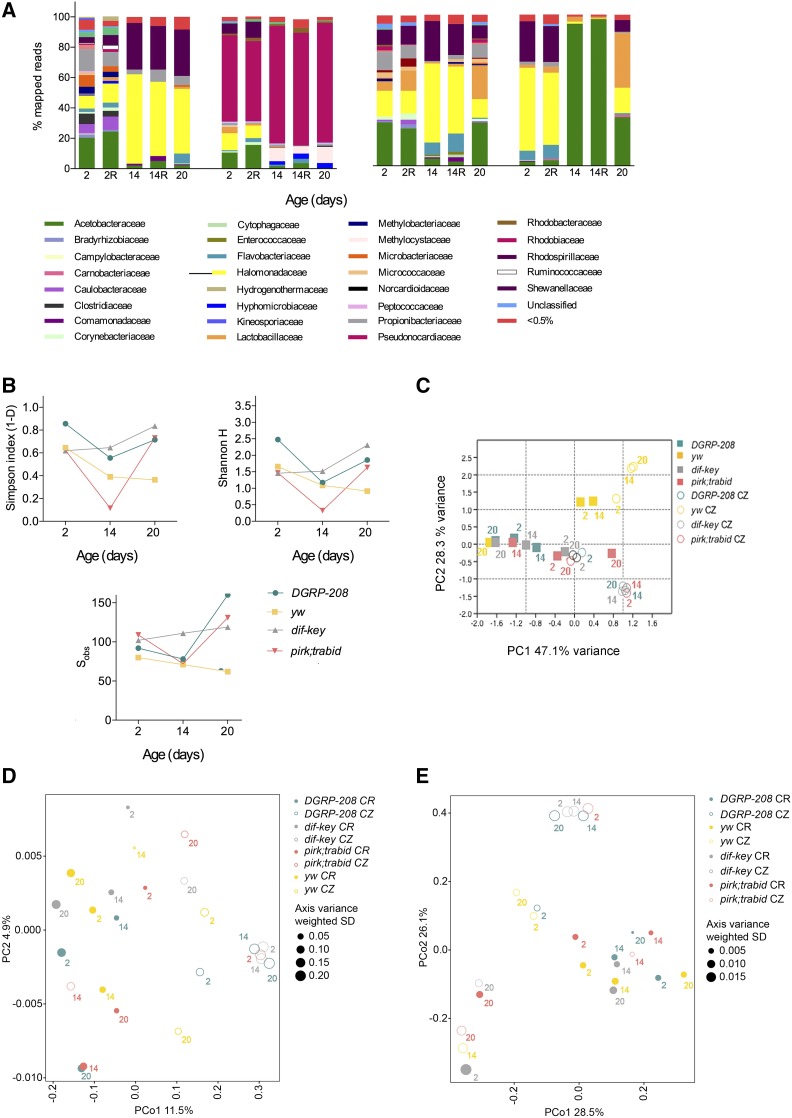
Microbiota of conditionalized (CZ) isolated guts. (A) The relative abundance of bacterial families detected in dissected guts from CZ flies in two controls (*DGRP-208* and *yw*) and mutant strains (*dif-key* and *pirk*;*trbd*) across life span as revealed by 16S rRNA sequencing. (B) α diversity indices (Simpson’s and Shannon H indices) and total number of observed families (S_obs_) for the four strains at selected time points (days 2, 14, and 20). (C) Principal Component Analysis (PCA) of the bacterial community of conventionally reared (CR) and CZ guts. (D) Principal Coordinate Analysis (PCoA) of unweighted UniFrac distances of the four strains in CR and CZ conditions. (E) PCoA of weighted UniFrac distances.

Applied statistical analysis included the following layers: (i) rarefaction analysis; (ii) *t*-tests (α diversity analysis) referring to Shannon H and Simpson a-indices to compare diversity between all the samples (Table S4); (iii) χ^2^ tests (β diversity analysis) based on the relative abundance of the microbiota composition between all the samples to statistically support differences in their microbiota composition (Table S5) (χ^2^ tests were all positive due to the overdispersed nature of our samples); (iv) Principal Component Analysis (PCA) (β diversity analysis) presented in plots in Figure 1C, Figure 2C, and Figure 5C; (v) unweighted UniFrac analysis presented by PCoA (β diversity analysis) (shown in Figure 1D, Figure 2D, and Figure 5D) to evaluate differences in microbiota composition between samples, taking into account the phylogenetic relationship between the bacterial families included in the microbiota of each sample; (vi) weighted UniFrac analysis presented by PCoA (β diversity analysis) (shown in Figure 1E, Figure 2E, and Figure 5E) was employed to evaluate differences in microbiota composition by taking into account both the phylogenetic relationship between the bacterial families as well as the relative abundance of each family in each sample’s microbiota; and (vii) factors-explained separation tests on the pairwise unweighted and weighted UniFrac distance matrices, including adonis, anosim, and permanova tests (Table S6), on PCoA plots of Figure 1, D and E to statistically support the categorical variable of hyperactive immunity in shaping the gut microbiota. The above analysis was not plausible for the rest of the PCoA plots due to the insufficient sample size of the compared groups.

#### Rarefaction analysis:

Rarefaction curves illustrating sample saturation were generated using Analytic Rarefaction v1.3 (http://www.uga.edu/∼strata/software/index.html).

#### α and β diversity analysis:

Analyzing the α and β diversity enabled us to decipher the structure of the gut microbiota composition further. Whittaker defined three terms for measuring biodiversity: α, β, and γ diversity (Whittaker 1972). He described α diversity as the diversity of a particular area, or in this case a community, expressing it as the number of species in that community (species richness). Several estimators of α diversity are commonly used in microbial community analysis, including Simpson index (1-D), Shannon H index, and number of observed families (S_obs_). These were employed in this investigation to analyze the *Drosophila* gut microbial community. Both the Simpson’s and Shannon H indices are measures of diversity taking into account species richness and evenness. Shannon H considers all families equally, while Simpson’s Index is highly dependent on the dominant families and a few rare families do not affect the index. The S_obs_ in each strain at a given time point is also given and includes families present at <0.5%. However, it is important to note that the α diversity considers only the number of individuals of bacteria and does not take into account the type of bacteria to which they belong. Thus, when comparing between communities, in this case between fly strains, speculations can only be made based on the abundance of individual bacteria and not the type of bacteria to which they belong. To consider this, β diversity was required. Whittaker defined β diversity as the difference in species diversity between communities (Whittaker 1972). A measure of this is the pairwise χ^2^ test that determines the significance of separation between two metagenomic samples. Additionally, PCA was applied that quantifies and visualizes the dissimilarity between samples by taking into account the relative abundance of each bacterial family when comparing between communities. It uses a linear transformation to convert potentially correlated variables into a set of values called principal components. Principal component 1 and 2 are plotted along the *x*- and *y*-axis, respectively, and represent the first- and second-highest variance. The position of each data point explains this variance in the context of the experimental parameter. Clustered data points therefore represent that the phenotypic variable (relative abundance of bacteria families) are common among these samples (Pearson 1901). β diversity was taken a step further by creating PCoA plots. This analysis is similar to PCA plots; however, it utilizes the phylogenetic distances between bacterial families of each sample. The distances were determined by unweighted and weighted UniFrac metrics. Unweighted UniFrac metrics is based solely on the evolutionary relatedness while weighted UniFrac metrics also incorporates the relative abundance of the bacterial families (Lozupone and Knight 2005). This is important, for example, when considering two or more samples consisting of different bacterial families. These families may be evolutionally closely related and thus not so distinct from each other. PCA and PCoA plots were both employed in this study to compare between different gut microbiota communities of the four *Drosophila* strains at various time points as it permits the simplistic visualization of data by reducing complex microbial communities to a single data point. γ diversity is the overall diversity for different ecosystems within a region but is not used for microbiome analysis (Whittaker 1972).

#### Software and statistical packages:

GraphPad Prism software (version 6) was used to design graphs and perform statistical analyses. Significance of QPCR for bacterial load analysis and gene expression and morphological analysis was determined using a paired Student’s *t*-test.

α diversity (Simpson 1-D, Shannon H, and S_obs_) and β diversity (PCA) diversity statistics were analyzed using PAST3 software (Hammer *et al.* 2001).

For β diversity UniFrac analysis, 25 reference sequences from the Greengenes database with known orientation to infer read orientation were used. UCLUST (in optimal mode) was used to carry out within-sample preclustering at 98% sequence identity, and representatives of each cluster were extracted. QIIME was used to perform open-reference OTU picking and taxonomy was assigned. PyNAST and FastTree were used to build the phylogenetic tree based on the cluster centroids. To complete the UniFrac analysis, the OTU table was subsampled without replacement 1000 times at 30 K observations per replicate. These subsamples were used to calculate weighted UniFrac estimates. Multiple PCoA ordination at 30 K observations per subsample was performed and the results were collated to estimate the standard variation of the sample position in the PCoA coordinate system. The results were visualized via a series of plots. The point size demonstrates axis variance-weighted SD in position. Adonis, anosim, and permanova tests were applied by using package vegan for the R programming language.

### Bacterial load analysis

#### Bacterial counts in whole flies:

At the selected time points during adult life span (days 7, 20, and 40), a single fly (*n* = 6) was washed in 70% ethanol for 2 min and further rinsed in sterile water prior to homogenizing using micropestles in MRS broth (69966, Sigma). Serial dilutions were plated on nutrient agar (MRS) plates (69964, Sigma) and incubated at 30°. Colonies were counted after 72 hr.

#### QPCR analysis in isolated guts:

QPCR was used to measure mRNA expression. Total RNAs were extracted using a Total RNA Purification Plus Kit (Norgen – Biotek, Canada) from five independent samples, each consisting of 20 midguts at the selected time points (days 2, 7, 20, and 40). cDNA was prepared from 0.5 μg total RNA using a Maxima First Strand cDNA Synthesis Kit (K1671, Thermo Fisher Scientific). The following primers were used for QPCR amplification of antimicrobial peptides (AMPs): attacin A (F: 5′-CTCCTGCTGGAAAACATC-3′; R: 5′-GCTCGTTTGGATCTGACC-3′), attacin D (F: 5′-AGTGGGGGTCACTAGGGTTC-3′; R: 5′-GTGGCGTTGAGGTTGAGATT-3′), and diptericin B (F: 5′-AGGATTCGATCTGAGCCTCAACGG-3′; R: 5′-TGAAGGTATACACTCCACCGGCTC-3′). To determine the gene expression of upd3 and socs36E, the following primers were used: upd3 (F: 5′-CTGGTCACTGATCTTACTCGCC-3′; R: 5′-GGATTGGTGGGATTGATGGGA-3′) and socs36E (F: 5′-ATGGGTCATCACCTTAGCAAGT-3′; R: 5′-TCCAGGCTGATCGTCTCTACT-3′). rp49 was used as the internal control: (F 5′-CCAGTCGGATCGATATGCTAA-3′; R: 5′-GTTCGATCCGTAACCGATGT-3′).

### Gut physiology

#### Enterocyte cell death:

Individual guts from CR and CZ *Drosophila* at selected time points (third instar larvae, day 2 and day 20 adults) (*n* = 20) for four strains (*DGRP-208*, *yw*, *dif-key* and *pirk;trbid*) were dissected in 1× PBS and immediately placed in 4% formaldehyde fixative for 15 min. Guts were washed twice in 1× PBS for 15 min each time then incubated in 5 × 10^−5^ mM Sytox green (S7020, Thermo Fisher Scientific) diluted in 1× PBS for 15 min on a rotating platform in the dark. Guts were twice washed in 1× PBS for 15 min. Samples were mounted on slides in 20% glycerol, visualized at magnification of ×20 on Axioplan 2 (Zeiss [Carl Zeiss], Thornwood, CA), and captured using AxioCam (Zeiss). The number of dead enterocytes was measured by counting the number of Sytox-positive cells in midguts (*n* = 20) of 7.5-mm^2^ regions using the measure functions within AxioCam software (Zeiss).

#### Morphological analysis:

Individual adult guts (*n* = 20) were dissected at selected time points (days 7, 20, and 40) in 1× PBS and immediately placed in 4% formaldehyde fixative for 15 min. Guts were then washed twice in 1× PBS, for 15 min each time, and then incubated in 1/1000 dilution of 5 mg/ml of DAPI for 30 min on a rotating platform in the dark. Guts were twice washed in 1× PBS for 15 min. Samples were mounted on slides in 20% glycerol, visualized on Axioplan 2 (Zeiss) at ×10 and ×20 magnification, and captured using AxioCam (Zeiss). Measurements to the nearest micrometer were obtained using the measure functions within AxioVision software (Zeiss). Length was measured by tracing from the middle of the proventriculus along the midgut to the hindgut. Enterocyte internuclei distance was determined by calculating the distance between large nucleus–DAPI-positive cells (*n* = 60) in three regions (foregut, midgut, and hindgut) of individual guts (*n* = 20 – 30).

#### Measuring internal gut pH:

Two pH indicator dyes, bromocresol purple (BCP) (114375, Sigma) and bromophenol blue (BPB) (B0126, Sigma), were used to determine the internal gut pH. The staining for BCP is purple at pH > 6.8 and yellow at pH < 5.2. The staining for BPB is yellow at pH < 3 and blue at pH > 4.6. Both dyes were prepared to 0.5% in 10% sucrose. Two hundred microliters of dye was added to whatman filter paper (Z274852, Sigma) placed in empty vials. Adult flies at selected time points (days 7, 20, and 40) were starved for 1 hr prior to feeding dye. Twenty flies were added per vial with dye for 1 hr. Guts were dissected immediately in 1× PBS. Dissected guts were visualized and captured on a Leica EZ4 HD stereomicroscope at magnification of ×10.

### Data availability

*Drosophila* strains are available upon request. All primary data can be found in Table S1. A complete list of statistical tests can be found in Table S3, Table S4, and Table S5. A full list of UniFrac Distances is included in Table S6.

## Results

Numerous studies have now been conducted to identify commensal bacterial species in the *Drosophila* gut (Ryu *et al.* 2008; Chandler *et al.* 2011; Storelli *et al.* 2011; Wong *et al.* 2011; Broderick *et al.* 2014). These studies revealed the simplicity of bacterial communities associated with the *Drosophila* gut compared to humans or mice in both laboratory strains and wild-caught flies. These communities belonged predominantly to the phylum of Firmicutes represented by the families of Lactobacillaceae and Enterococcaceae and the phylum of Proteobacteria represented by the families of Acetobacteraceae and Enterobacteriaceae. From these families, dominant individual species were those of *Lactobacillus plantarum*, *L. brevis*, *Acetobacter ponorum*, *A. pasterianus*, and *Enterococcus faecalis* (for review see Erkosar *et al.* (2013)).

We employed high-throughput NGS to determine the potential influence of immune status on the structure and relative abundance of bacterial species residing in the gut across the life span. We oversaturated our sequencing reads to remove among-sample differences. Following quality filtering (see *Materials and Methods*), all rarefaction curves tended to saturation (Figure S1). This indicated that all OTUs were representative of the total bacterial community of each sample. For a description of our experimental set-up and biological repeats see *Materials and Methods*. To see results on relative abundance and comparisons to repeats see Figure 1 and Figure 2. For PCoA plots comparing original experiments and repeats see Figure S2 and Figure S3.

### Comparisons between CR and CZ flies

In CR flies, we detected 15–20 bacterial families common to all fly strains but the total number of bacterial families detected within each strain varied from 30 to 100 families (Figure 1A and S_obs_ index in Figure 1B). Of note, the number of cultivatable bacteria was much smaller than the total number of molecularly identified families (Table S1 and Table S2). For all fly strains, we observed a reduction in diversity with age coupled to Acetobacteraceae expansion (Figure 1, A and B). Lactobacillaceae, another dominant strain found in CR *Drosophila*, was also present in all four strains; however, not to the same extent as Acetobacteraceae. In addition, there was an age-dependent increase in bacterial load measured as indicated by CFUs across the life span of all four strains in accordance with previous literature (Wong *et al.* 2011; Figure S4A) as well as determined by molecular quantification of the V3 region of the 16S rRNA gene by QPCR across the life span of all four strains (Figure S4B).

In CZ flies, a statistically significant increase in gut microbiota diversity in all fly strains compared to their CR self was revealed (Figure 2 and Table S4 for diversity *t*-tests). For example, pairwise comparisons at day 20 between Shannon H and Simpson indices of diversity in CR and CZ conditions resulted in *P*-values between 0.0008 (*pirk;trbd*), 0.0003 (*dif-key*), and <0.0001 (*yw*, *DGRP-208*) including their biological (day 20) CR repeats. Specifically, Acetobacteraceae and to a lesser extent Lactobacillaceae were replaced by other bacterial families in the gut of *yw* and *DGRP-208* (compare Figure 1, A and B; see below). Familial transmission of these bacteria therefore blocked diversity and made the gut microbiota structure simpler in CR conditions (see Table S4 for comparisons between CR and CZ). In contrast to *yw* and *DGRP-208*, Acetobacteraceae and Lactobacillaceae still remained an important part of the microbiota of the two mutant strains. Diversity patterns revealed that, in CZ flies, *dif-key* was the most diverse strain of the four (Figure 2B and Table S4 for statistics of pairwise comparisons). Pairwise comparisons for β diversity in both CR and CZ flies measured through χ^2^ did not give meaningful results since all comparisons were found to be significant (Table S5). This indicated that the samples were overdispersed and instead, factorial β diversity tests were used to compare UniFrac analyses (Table S6).

Bacterial density in the gut of all young (5-day-old) CZ flies was significantly reduced in relation to their CR counterparts (Figure S4C). Moreover, during the time of observation CZ flies had consistently lower bacterial loads than their CR counterparts (Figure S4C). This showed that the main contributor of bacterial density in CR flies was familial transmission. However, a notable exception was *yw* CZ, which in 2 weeks obtained the same density levels as *yw* CR (Figure S4C).

Further analysis at the level of genus for the most abundant families revealed that, under CR conditions, the most representative bacterial genera were *Acetobacter* and *Lactobacillus*, while *Acidimonas*, *Corynebacterium*, and *Shewanella* followed at high levels (Figure S5). By removing competition from familial transmitted bacteria, genera such as *Shewanella* and those belonging to the family of Rhodobiaeae were able to thrive. *Acetobacter* and *Lactobacillus* remained detectable but at significantly lower levels (Figure S5).

Finally, culture-dependent experiments (Table S1) identified *L. plantarum*, *L. pentosus*, and *A. pasteurianus*, as the dominant species of Lactobacillaceae and Acetobacteraceae during the life span. As shown previously, *L. plantarum* has a stable association with the *Drosophila* gut (Storelli *et al.* 2011).

### Comparisons in gut microbiota of wild-type and control strains

Relative abundance measurements (Figure 1A) coupled with Shannon H index analysis (Figure 1B) indicated that, between them, *yw* and *DGRP-208* differed greatly in the diversity of their intestinal microbiota. Unlike the Simpson’s index, which is largely influenced by the dominant bacterial families, the Shannon H index considers all bacterial families equally and is affected by families with few representatives. Although both *DGRP-208* and *yw* followed the same trends in their gut microbiota across the life span, *DGRP-208* was a more diverse strain bearing a number of bacterial families (with low representation) that were not found in *yw*. Thus, the diversity of *DGRP-208* across the life span was greater than that of *yw* (reflected in the *t*-test results of the Shannon H and Simpson index, see Table S4). For example, comparing diversity between *DGRP-208* and *yw*, in CR conditions: day 2 Shannon H *P*-value = 0.0758, Simpson *P*-value = 0.0101; day 7 Shannon H *P*-value = 0.0738, Simpson *P*-value = 0.4274; day 7 Repeats Shannon H *P*-value = 0.0623, Simpson *P*-value = 0.4821; day 14 Shannon H *P*-value = 0.0738, Simpson *P*-value = 0.4274; day 20: Shannon H *P*-value = 0.2019, Simpson *P*-value = 0.4484; and day 20 Repeats: Shannon H *P*-value = 0.1242, Simpson *P*-value = 0.4272). This was in accordance with the increased number of bacterial families shown in S_obs_ (Figure 1B). However, on day 40 PE, both strains showed statistically indistinguishable α diversity indices (Shannon H *P*-value = 0.9447 and Simpson *P*-value = 0.8468), as was evident from the dominance of *Acetobacter* in their gut microbiota. Therefore, the α diversity analysis showed that *DGRP-208* had a more diverse microbiota structure.

To compare dissimilarities between the microbiota of the two fly strains, we employed PCA (taking into account relative abundance, Figure 1C) as well as unweighted (taking into account phylogenetic relationship, Figure 1D) and weighted (taking into account both relative abundance and phylogenetic relationship, Figure 1E) PCoA. These analyses showed again that *DGRP-208* and *yw* were very different at early ages (UniFrac distances for day 7, 0.8080 unweighted and 0.6098 weighted) but both converged in old age (UniFrac distances for day 40, 0.7473 unweighted and 0.4260 weighted). CFUs for both fly strains showed that they had the same levels of cultivable bacteria across the life span (Figure S4A). Nevertheless, there was an increase in bacterial load for both when compared to their 7-day self (Figure S4B). Moreover, QPCR analysis confirmed that these two fly strains followed the same trend/progression in bacterial load across their life span (Figure S4C).

In CZ flies, bacterial families that dominated the microbiota in CR conditions in both the *yw* and *DGRP-208* strains, namely Acetobacteraceae and Lactobacillaceae, were replaced by others (Halomonadaceae and Shewanellaceae in DGRP-208 and Rhodobiaceae in *yw*) (Figure 2A). This strongly suggested that Acetobacteraceae and Lactobacillaceae were mainly present due to familial transmission. CZ *yw* flies presented an age-dependent reduction in diversity as seen in Simpson’s and Shannon’s H indices (Figure 2B). Nevertheless, both CZ fly strains were more diverse compared to their CR selves as seen in α diversity measurements of *t*-tests for both Shannon H and Simpson indices (Table S4). In addition, the statistically significant degree of dissimilarity in their CZ microbiota compared to their CR selves was shown in β diversity PCA (Figure 2C) and PCoA plots (Figure 2, D and E). There, CZ *yw* and *DGRP-208* clustered away from their respective CR time points presenting greater UniFrac distances between these points than between CZ *yw* and CZ *DGRP-208* (Table S4). For example, the unweighted UniFrac distance between 20 days CZ *yw* and its CR self was 0.9023, whereas between 20 days CZ *yw* and CZ *DGRP-208* was 0.8277. The weighted UniFrac distance between 20 days CZ *yw* and its CR self was 0.7708, whereas between 20 days CZ *yw* and CZ *DGRP-208* was 0.6211. Therefore, at 20 days, gut microbiota diversity was closer between CZ flies of *DGRP-208* and *yw* than between CZ and CR flies of the same strain.

To compare the two fly strains in CZ conditions, we took into account relative abundance and α diversity *t*-tests of *DGRP-208* and *yw* (see Table S4). We found that *yw* was consistently less diverse than *DGRP-208* across the life span in CZ conditions (*e.g.*, day 2 and day 2 repeats *P* < 0.0001 for both Shannon H and Simpson indices; day 20, *P* = 0.0007 for Shannon H and *P* < 0.0001 for Simpson), reflecting what was found in CR conditions and indicating an intrinsic difference in the two fly strains based on the genetic background.

### Gut microbiota in immune-compromised flies

*Dif-key* CR guts displayed an early dominance of *Lactobacillus* that gave way to a gradual age-dependent increase in Acetobacteraceae, which following day 20 resembled a mono-association (>90%; Figure 1A). When each *dif*;*key* CR time point was compared to the corresponding *yw* CR time point, microbiota diversity of *dif-key* flies closely followed their genetic background across the early- and midlife span but differentiated from *yw* at day 40 (Figure 1B and Table S4). Specifically, α diversity *t*-tests for Shannon H and Simpson indices with respective *P*-values 0.7908 and 0.4493 for day 2; 0.1089 and 0.7273 for day 7; 0.2126 and 0.6528 for day 7 repeats, 0.8431 and 0.0190 for day 14; 0.3250 and 0.1263 for day 20; 0.3650 and 0.2165 for day 20 repeats; and <0.0001 for day 40. Interestingly, both 20- and 40-day-old *dif-key* CR sibling flies resembled each other indicating that their gut microbiota did not undergo any significant changes from middle age onwards (Figure 1, A–C). Unweighted β-diversity metrics (taking into account phylogenetic relationship) confirmed that the microbiota of 20- and 40-day-old *dif-key* CR sibling flies had the smallest UniFrac distance (0.7648) between each other than with any other time point/genotype (Figure 1D and Table S6). In their turn, both of these *dif-key* time points resembled the 20-day-old *yw* genetic background (Figure 1C). Weighted β diversity metrics (taking into account both relative abundance and phylogenetic relationship) indicated that 40-day-old *dif*;*key* flies had the smallest UniFrac distance (0.1854) with 20-day-old *yw* (Figure 1E and Table S6). This further underlined the lack of development with age in the microbiota structure of an immune-compromised gut, where Acetobacteraceae speedily took over the bacterial landscape. As expected, *dif-key* had a higher bacterial load compared to the *yw* control (Figure S4).

By removing maternally transmitted bacteria, relative abundance and diversity metrics (Simpson and Shannon indices) showed that the microbiota of CZ *dif*;*key* flies was more diverse than their *yw* background accompanied by a high number of distinct bacterial families (Figure 2B). Furthermore, the previous Acetobacteriaceae family domination was now replaced by bacteria belonging to the genus of *Shewanella* and genera representing the Halomonadaceae family (Figure 2A and Figure S5). Interestingly, following β diversity analysis (Figure 2C), microbiota patterns of *dif-key* CZ flies were found to be different from their genetic background. Specifically, unweighted UniFrac distances between CR *yw* and CR *dif-key* were 0.7633, 0.7829, and 0.7681 at 2, 14, and 20 days PE, respectively, whereas the same distances in CZ conditions were 0.7770, 0.8614, and 0.8888 (Table S4). Similar conclusions were drawn when weighted UniFrac distance were examined with distances between CR *yw* and CR *dif-key* at days 2, 14, and 20, *i.e.*, 0.5504, 0.2995, and 0.3944, while in CZ conditions the same distances were 0.3999, 0.7976, and 0.5822 (Table S4). This revealed that familial transmission was dominant over the lack of immunity to shape the gut microbiota in CR conditions. When familial transmission was removed, the influence of a deficient immune system was highlighted (see *Discussion*).

### Gut microbiota in a constitutively active immune system

Bacterial gut populations in *pirk;trbd* had a different dynamic across the life span compared to its genetic background, as indicated by relative abundance measurements (Figure 1A).

Diversity indices of CR guts showed that *pirk;trbd* flies started with a diversity comparable to all other fly strains (Figure 1B). However, at day 7, a consistent pattern of low diversity ensued. This pattern continued to middle age (day 14) where *pirk;trbd* gut microbiota were dominated by two bacterial families, namely Corynebacteriaceae and Acetobacteraceae, covering >95% of relative abundance. This resulted in low diversity compared to both *yw* and *dif-key* (see Simpson and Shannon H indices in Figure 1B and Table S4). On day 20 PE (very near the end of the strain’s median life span), the dominant gut OTU was Enterococcaceae (Figure 1A), represented mainly by bacteria belonging to the genus of *Enterococcus* (Figure S5).

Overall, the intestinal microbiota of both CR (Figure 1C) and CZ (Figure 2C) *pirk;trbd* flies were significantly different in terms of structure from their genetic background (see below). In addition, microbiota composition of *pirk;trbd* CR flies was also different compared with *dif-key* (Figure 1C). This indicated an effect of their immune status over and above familial transmission. To strengthen this conclusion, we applied factors-explained separation tests (adonis, anosim, and permanova tests) for both weighted and unweighted UniFrac distances. We grouped all CR *pirk;trbd* samples and compared them against all CR *yw* samples, asking if hyperactive immunity resulted in statistically distinguishable gut microbiota compared with control flies. Results gave significant differences for all tests applied (adonis test *P*-value = 0.03992, anosim test *P*-value = 0.06205, and permanova test *P*-value = 0.05433). In other words: all three tests showed significant probability of nonrandom separation of unweighted UniFrac distances (Figure 1D and Table S6). However, this was not the case when comparing *dif-key* *vs.* *yw* or *DGRP-208* *vs.* *yw* (Figure 1D and Table S6). Moreover, using CR biological repeats (Figure S2) we obtained similar results (adonis *P*-value = 0.02842, anosim *P*-value = 0.052877, and permanova *P*-value = 0.05821), which confirmed the role of hyperactive immunity, and the consistency of the results across different generations and over any seasonal fluctuations of microbiota (Wong *et al.* 2011). The statistically significant differences between CR *pirk;trbd* and *yw* revealed in unweighted UniFrac distances were not presented when weighted UniFrac distance were measured (Figure 1E and Table S6). The latter analysis takes into account relative abundance not computing equally, therefore rare and major OTUs. This meant that differences observed in the unweighted UniFrac analysis were mostly due to differences in minor components of the community (see *Discussion*). In addition, *pirk;trbd* CR and CZ had a variable but consistently high bacterial load across the life span, in contrast to all other strains where there was an age-dependent increase of bacterial load (Figure S4).

Taken together, our results indicated that flies with a constitutively active immune system had a structure and density of gut microbiota that differed both from their genetic background and from immune-compromised flies. Moreover, persistence of Acetobacteraceae in CR *pirk;trbd* was also observed in CZ *pirk;trbd*. Therefore, this bacterial family was not outcompeted by other bacteria in CZ conditions as seen in the other fly strains (Figure 2A). Thus, constitutively active immunity may result in the acceleration of the Acetobacteraceae-dominated ageing pattern in microbiota composition.

*Pirk-trbd* CZ flies showed diversity that was statistically indistinguishable from *dif-key* CZ and *yw* CZ flies at young age (2 days PE; Figure 2B). Specifically, respective *P*-values of *t*-tests for Shannon H and Simpson indices between *pirk;trbd* and *yw* were 0.5084 and 0.7315 (0.5986 and 0.6835 for the biological repeats) (Table S4). Previously present bacterial families in CR *pirk;trbd* were replaced by bacterial genera belonging to the families of Halomonadaceae and Swevanellaceae following similar trends as CZ *dif-key* flies (Figure 2A and Figure S5). Fourteen-day-old *pirk;trbd* CZ flies showed lower diversity than both *dif-key* and *yw* flies represented by Acetobacteraceae domination (Figure 2, A and B and Table S4). This was much like the expansion seen in *pirk;trbd* CR (Figure 1A). However, at the age of 20 days PE, *pirk;trbd* guts tolerated this *Acetobacter* domination and gained in diversity (Figure 2A and Table S4). PCA plots (see Figure 2C) showed that, as they aged, the degree of dissimilarity between CZ *pirk;trbd* and CZ *dif-key* was less than the dissimilarity between each one of them and their *yw* genetic background. For example, unweighted UniFrac distance between CZ *pirk;trbd* and CZ *dif-key* at day 20 was 0.7496, while the same distance between CZ *pirk;trbd* and CZ *yw* was 0.8792 and between CZ *dif-key* and CZ *yw* was 0.8355 (Figure 2D and Table S6). Weighted UniFrac distances confirmed this with the distance between CZ *pirk;trbd* and CZ *dif-key* at day 20, at 0.3395; the same distance between CZ *pirk;trbd* and CZ *yw* was 0.7980 and between CZ *dif-key* and CZ *yw* was 0.5822 (Figure 2E and Table S6). Therefore, when familial transmission was eliminated, middle-aged flies from immune-aberrant strains resembled each other more than their genetic background. Moreover, removing familial transmission extensively changed the age-dependent dynamics of the gut microbiota structure and diversity in *pirk;trbd*, indicating that familial transmission strongly interacted with immune status in defining the bacterial landscape of the gut in these flies. Nevertheless, CZ *pirk;trbd* flies were more similar to their CR self than CZ *yw* (unlike *DGRP-208* and *yw* for example). Specifically, PCA plots showed that both *yw* CR and CZ flies clustered away from *pirk;trbd* CZ (Figure 2C). In addition, weighted UniFrac distances between CZ *pirk;trbd* flies and their CR self was 0.6977 while the one between CZ *pirk;trabid* and CZ *yw* flies was 0.7980 (Figure 2E and Table S6). Therefore, even when familial transmission was removed, the significant differences between *pirk;trbd* and the *yw* genetic background remained.

### Immune status and gut physiology

Since microbial colonization depends on the physical and chemical conditions of the different gut compartments, we checked several factors involved in gut physiology. Applying two different dyes (BCP, purple at pH 6.8 and yellow at pH 5.2 and BPB, yellow at pH < 3 and blue at pH > 4.6), we detected no differences in adult gut pH at various points across the life span (7, 20, and 40 days PE) (Figure S6 and Figure S7). Additional features including gut length (Figure S8A) and gut internuclei distance (foregut, midgut, and hindgut) (Figure S8, B–D, respectively) were checked in all fours CR strains and again showed no difference.

**Figure 3:**
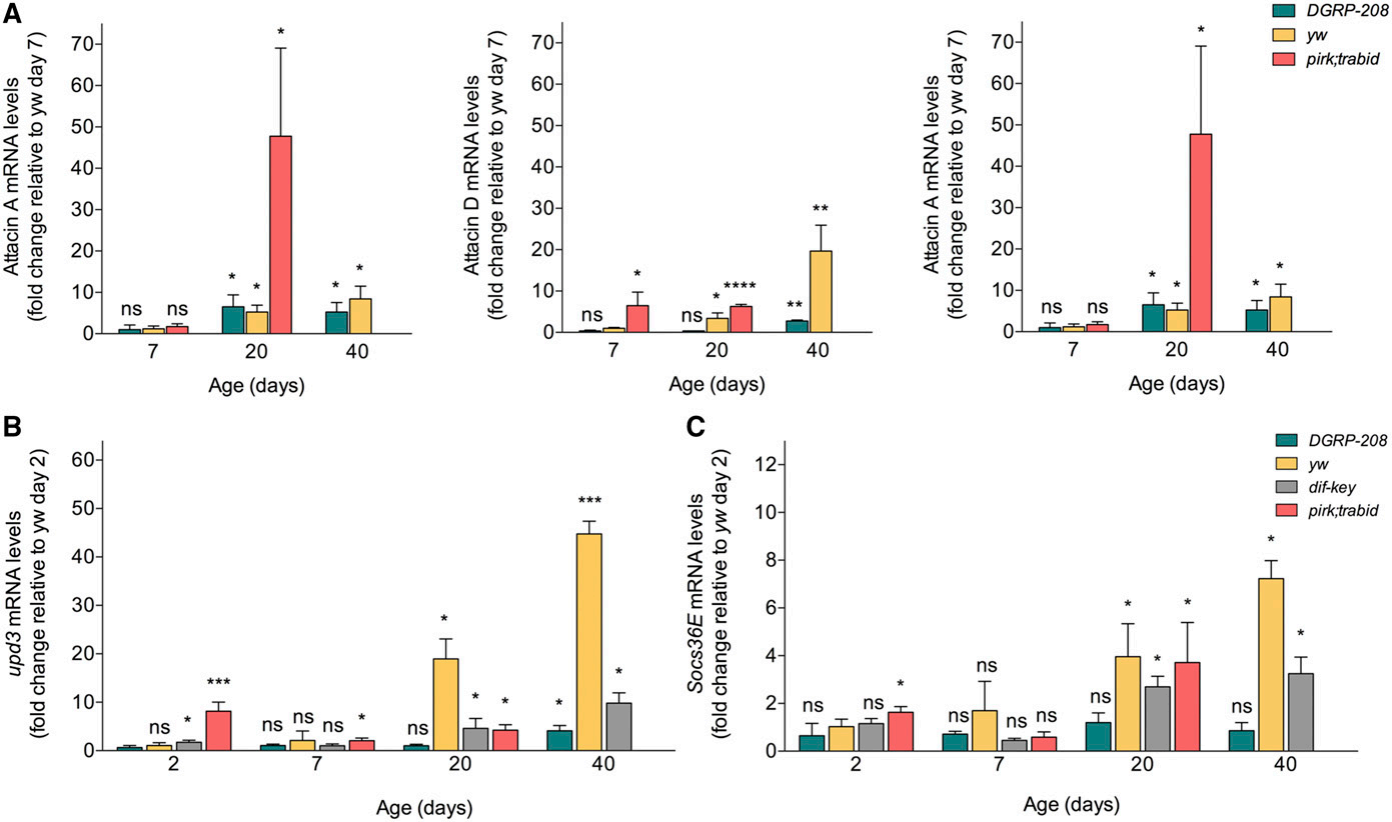
Immune status and JAK-STAT activity. (A) mRNA gene expression of three AMPs (attacin A, attacin D, and diptericin B) with age in the four fly strains (*DGRP-208*, *yw*, *dif-key*, and *pirk*;*trbd*). Error bars represent the SD of five separate experiments; ns, *P* > 0.05, *P < 0.05, **P < 0.01, ***P < 0.001, and ****P < 0.0001 indicate significance value when compared to *yw* day 7. (B) Upd3 mRNA gene expression in all four strains across life span. Error bars represent the SD of three separate experiments; ns, *P* > 0.05, *P < 0.05, **P < 0.01, and ***P < 0.001 indicate significance value when compared to *yw* day 2. (C) Socs36E mRNA gene expression in four strains with age. Error bars represent the SD of three separate experiments; ns, *P* > 0.05, *P < 0.05 specify significance value when compared to *yw* day 2. ns, not significant.

**Figure 4:**
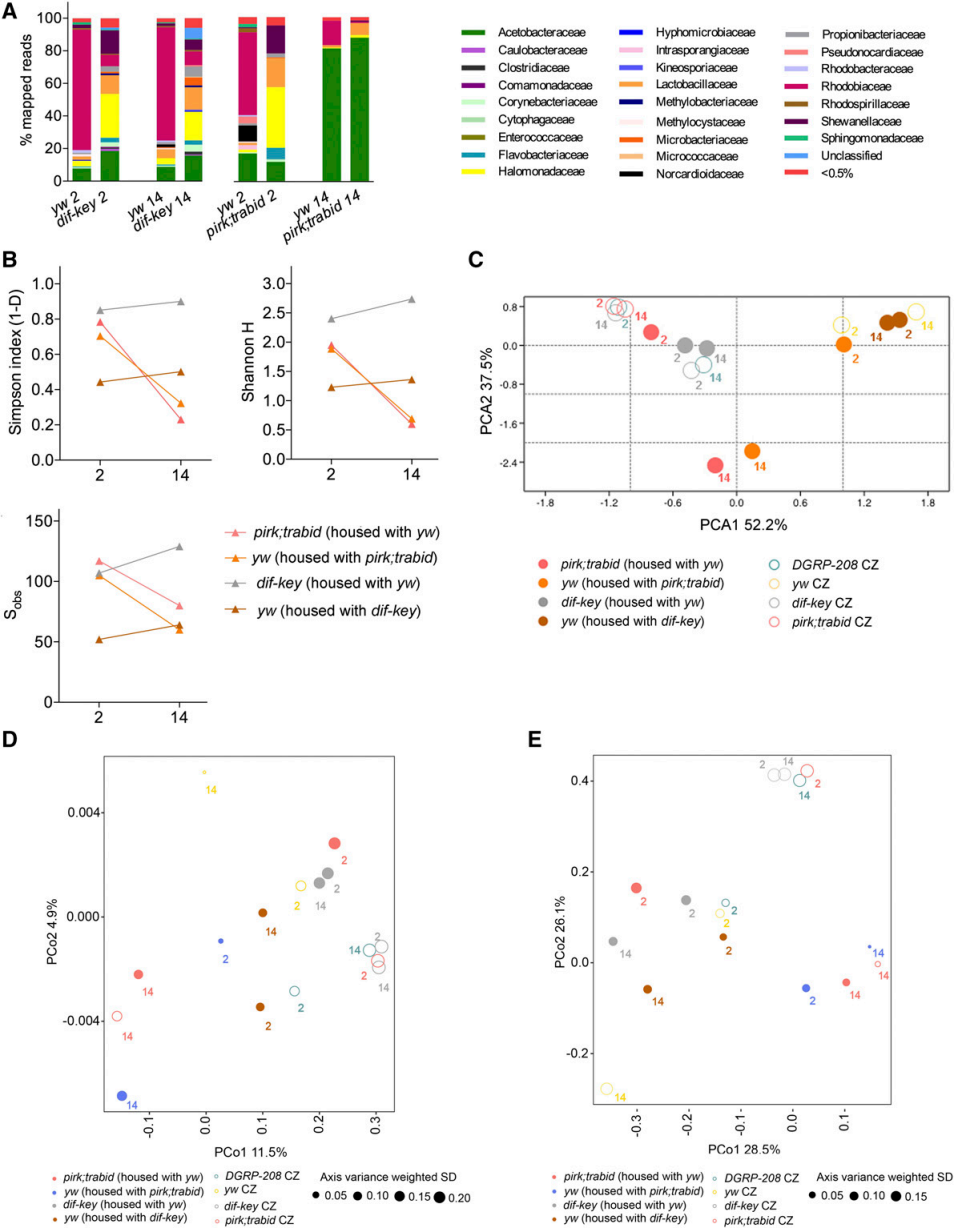
The effect of cohousing on gut microbiota composition. (A) The relative abundance of bacterial families detected in dissected guts of cohoused strains (*yw* and *dif-key*; *yw* and *pirk;trbd*) disclosed by 16S rRNA sequencing. (B) α diversity indices (Simpson’s and Shannon H indices) and total number of observed families (S_obs_) for the four strains at selected time points (day 2 and 14). (C) Principal Component Analysis (PCA) of the bacterial community of cohoused strains and corresponding CZ strains. (D) Principal Coordinate Analysis (PCoA) of unweighted UniFrac distances of the cohoused strains and corresponding strains in CZ conditions. (E) PCoA of weighted UniFrac distances. CR, conventionally reared; CZ, conditionalized; ns, not significant.

Predictably, AMP gene expression levels were markedly increased in *pirk;trbd* flies compared to *yw* throughout their life span, despite the fact that there was an age-dependent increase in AMP transcription in all fly strains (Figure 3A). This indicated an inflamed gut with stressed enterocytes, which in turn implied increased stem cell activity. Indeed, we found a statistically significant increase of early (2 and 7 days) gene expression in *pirk;trbd* for the cytokine upd3, which triggers Janus kinase signal transducer and activator of transcription (JAK-STAT) signaling in stem cells (Figure 3B). This reflected an early increase of JAK-STAT activity measured by gene expression of the JAK-STAT pathway target *socs36E* (Figure 3C). Furthermore, there was a statistically significant difference in enterocyte cell death in *pirk;trabid* guts, as detected by the vital dye Sytox green across the life span in both larvae and adults (Figure 4A for images and Figure 4B for quantification). Increased AMP levels and high cell death was accompanied by an increase in bacterial load across the life span of *pirk;trbd* (Figure S4).

All CZ strains exhibited reduced cell death in comparison to CR guts (see Figure 4A for images and Figure 4B for quantification). Specifically, CZ conditions lowered gut cell death in very low levels in larvae while the age-dependent increase in gut cell death observed in CR adults was also significantly reduced (Figure 4B). This showed the importance of the microbiota in regulating gut homeostasis and revealed its major effect in cases of unregulated immunity (as in *pirk;trbd*), where inflamed conditions were promoted by the presence of bacteria in the gut.

### Constitutive immunity influences the local bacterial microenvironment

Familial transmission and food are the sources of bacteria for laboratory cultures of *Drosophila* (Wong *et al.* 2015). The contribution of familial transmission can be addressed by producing CZ flies. However, as recently shown, the host drives microbiota changes there through its constant interaction with food, which in turn alters the gut microbiota landscape (Wong *et al.* 2015). The microenvironment therefore plays an important role in shaping gut microbiota. But what is the influence of the immune status of flies on the microenvironment?

**Figure 5:**
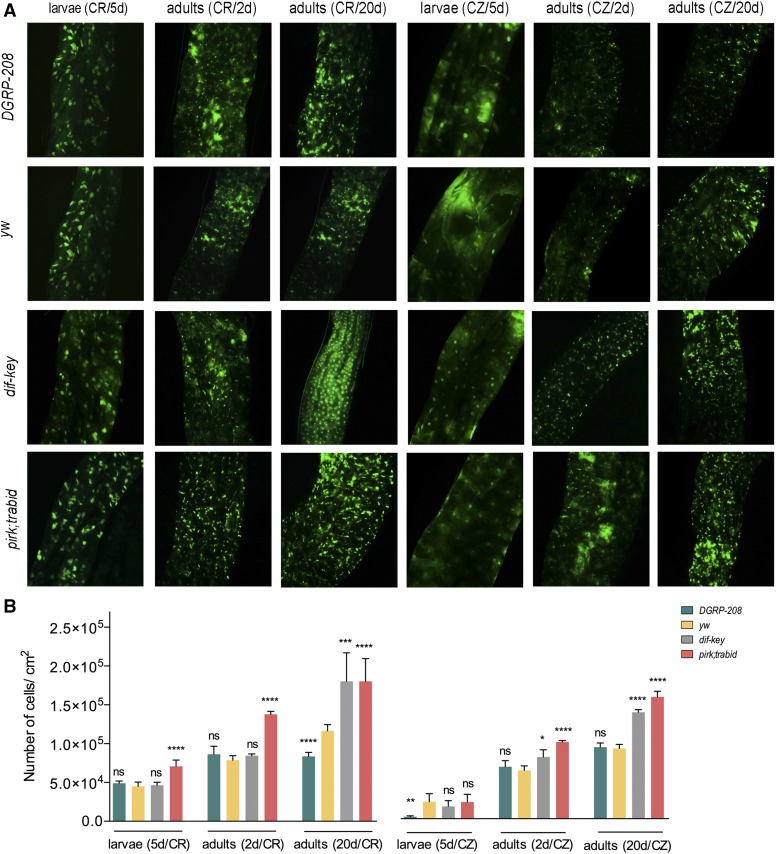
Enterocyte cell death in CR and CZ dissected guts. (A) Sytox green staining of the dissected guts from 5 day larvae, 2 and 20 day adults of the four strains (*DGRP-208*, *yw*, *dif-key*, and *pirk;trbd*) under CR and CZ conditions. (B) The number of dead cells of the above four strains at the above time points under CR and CZ conditions. Error bars represent SD of six separate experiments; ns, *P* > 0.05, *P < 0.05, **P < 0.01, ***P < 0.001, and ****P < 0.0001 indicate significant values when compared to *yw* of the corresponding time point. CR, conventionally reared; CZ, conditionalized; ns, not significant.

To find out whether microbiota differences in CZ flies could be influenced by immune status during co-culture, we mixed bleached fertilized eggs of *pirk-trbd* or *dif-key* flies with *yw*. Using high-throughput NGS, we determined the bacterial communities in guts after 2 weeks of co-culture (14 days PE) and compared them to CZ flies at 2 and 14 days of single culture (see Figure 5A). Of note, that crowding conditions in both single and co-culture were identical with a total of 30 flies in each vial. *Dif-key* flies retained constant levels of diversity and exhibited the same pattern of age-dependent increase of diversity as single cultured *dif-key* CZ flies (Figure 5B; see Simpson and Shannon H indices). Likewise, *yw* remained more comparable to their single culture than to *dif-key* when co-cultured with the latter (Figure 5C). PCoA plots showed that unweighted as well as weighted UniFrac distances were smaller between *yw* CZ and *yw* cohoused with *dif-key* than between *yw* cohoused with *dif-key* and their *dif-key* cohabitants (Figure 5, D and E and Table S6).

In contrast, co-culturing *yw* and *pirk;trbd* resulted in gut microbiota of *yw* being driven by *pirk;trbd* toward an almost mono-association with Acetobacteraceae and a complete absence of Lactobacillaceae (Figure 5A). Moreover, *yw* flies exhibited reduced diversity with age, thus resembling *pirk;trbd* (Figure 5B; see Simpson and Shannon H indices and compare to Figure 1B and Figure 2B). In addition, PCA indicated that by the end of co-culturing (14 days), the gut microbiota landscape of *yw* became more like their nonsibling *pirk;trbd* cohabitants (Figure 4C). PCoA plots showed that both unweighted and weighted UniFrac distances between *pirk;trbd* and *yw* cohabitants were less than distances of cohoused *yw* with their siblings in single culture (Figure 5, D and E and Table S1). Specifically, at 14 days, the UniFrac distances between *yw* and their *pirk;trbd* cohabitants was 0.1992 (weighted) and 0.7129 (unweighted), whereas with their *yw* CZ siblings (single culture) UniFrac distances were 0.6091 (weighted) and 0.7965 (unweighted). Taken together, these results showed that *pirk;trbd* had a statistically significant influence on *yw* microbiota through their common microenvironment.

## Discussion

### Immune-compromised flies and their genetic background have similar resident bacteria under conventional conditions

When comparing the diversity pattern of gut microbiota during the life span between CR flies that had a defective immune system (*dif-key*) and their *yw* genetic background, we found that they were very similar. The two strains had identical trends in the structure of their gut microbiota during their life span, with almost a mono-association with Acetobacteraceae at 20 days PE. However, absence of immunity led to the accelerated domination of Acetobacteraceae. In middle age *dif-key*, this domination was complete and persistent in older *dif-key* flies but not in old *yw* flies (40 days PE) that displayed a more varied gut microbiome. In this sense, our data suggested a pattern of aging for gut microbiota of immune-compromised flies. Specifically, the microbiota of *dif-key* in both 20 and 40 days PE were very similar to 20-day old *yw*, suggesting very little development of the bacterial population structure in the gut of *dif-key* beyond 20 days of age.

Removal of familial transmission revealed several differences between CZ *yw* and *dif-key* gut microbiota, indicating that the absence of a functional immune system affected the structure and diversity of OTUs in these conditions. This result showed that familial transmission prevented an underlying diversity that could be acquired through the interaction of the immune status with bacteria obtained from food.

In addition, and in contrast to *yw*, we observed a higher age-dependent gut microbiota density in *dif-key* flies. Therefore, as expected, an intact immune system was needed to regulate the number of gut bacteria. Our results indicated that under homeostatic conditions, the landscape of gut microbiota was primarily communicated by maternal transmission. Conversely, the impact of immune deficiency on the composition and diversity was only revealed when familial transmission was removed. This is in line with some of the evidence from TLR-deficient mice (Ubeda *et al.* 2012).

### Constitutively active immunity influences gut microbiota structure

In humans, chronic immune hyperactivity results in gastrointestinal infections, metabolic imbalances, and inflammatory bowel diseases, which can lead to colorectal cancer (Garrett *et al.* 2010). We used a *Drosophila* mutant (*pirk;trbd*), which mimics conditions of constant immune activity by derepressing the IMD pathway. *Pirk;trbd* CR flies had a microbiota with different diversity pattern, population structure, and bacterial density across the life span compared to *yw* or *dif-key* CR flies. As metrics of β diversity showed, *pirk;trbd* CR flies presented a higher degree of dissimilarity to their genetic background than when comparing *dif-key* flies with the same genetic background. However, this dissimilarity was observed only in unweighted and not in weighted UniFrac distances. This can be explained if we consider that unweighted distances take into consideration rare and common OTUs equally, whereas weighted distances take into account relative abundance so that a rare OTU does not have the same importance in assigning UniFrac distance as a common one. This is turn means that differences between *pirk;trbd* and *yw* flies were due to changes in rare components of the flora and the appearance of novel OTUs (such as Enterobacteriaceae).

Removal of familial transmission eliminated some of these novel OTUs from the microbiome of CZ *pirk;trbd* flies but metrics of β diversity analysis showed that *pirk;trbd* CZ flies were still more closely related to their CR siblings than to CZ *yw* flies. This indicated the influence of the immune system in *pirk;trbd* flies over and above familial transmission. Differences in the *pirk;trbd* microbiota resulted in increased gut cell death compared to CR *yw*. In CZ conditions, gut cell death was initially significantly decreased. This result showed that maternally-transmitted microbiota as well as immune activity were responsible for the integrity of the epithelium and predisposed flies to gut inflammation in *pirk;trbd*. A recent study has shown that alterations in the microbiota, both in load and composition, precede and predict the onset of intestinal barrier dysfunction in aged flies (Clark *et al.* 2015). Our data suggest that this process is greatly accelerated and enhanced in a context of constitutively active immunity rather than in an immune-deficient one.

In humans, Irritable Bowel Disease (IBD) has been linked with characteristic shifts in the composition of the intestinal microbiota, reinforcing the view that IBD results from altered interactions between intestinal microbes and the immune system [for review see Hyland *et al.* (2014)]. However, the direct and indirect link of various factors in addition to immunity, such as diet, age, and drug treatment, can be also important. Their relative importance is nevertheless unclear, since cause and consequence are intricately connected. In this context, a genetically tractable model such as *Drosophila*, with easy husbandry and highly streamlined genetic backgrounds, can be useful to isolate the contribution of a constitutively active immune system to the disease phenotype.

The fact that a constantly inflamed fly gut accumulated Enterobacteriaceae in general and *E. feacalis* in particular, accompanied by extensive cell death during the life span, fits data in mice and humans. There, endogenous inflammation-induced reactive oxygen species (ROS) produced by the host favor commensal accumulation of Enterobacteriaceae (Lupp *et al.* 2007). The latter then produce dangerous oxygen radicals, linked to sporadic colorectal cancers in mammals (reviewed in Seksik *et al.* (2006)). Recently, Dantoff and colleagues showed that flies mutant in *nubbin* (*nub*) showed higher microbiota levels characterized by the detection of increased quantities of Enterococcaceae (as in *pirk;trbd* flies; Dantoft *et al.* 2016). Transcriptome analysis of *nub^1^* flies also showed increased expression of genes involved in the production of ROS and in resistance to oxidative stress. More work is needed to distinguish between the sequence of events that lead to the altered microbiota and increased cell death in the *pirk;trbd* flies, but the above are clear indications.

Gut microbiota in humans has been studied extensively and the two dominant phyla present in healthy human guts include Firmicutes and Bacteroidetes, both mainly obligate anaerobes (Lozupone and Knight 2005; Turnbaugh *et al.* 2009a,b). However, in patients with IBD, an imbalance of the gut microbiota was observed, with a reduction in members from Firmicutes phyla, thus fewer species of obligate anaerobes (Seksik *et al.* 2006; Swidsinski *et al.* 2007; Manichanh *et al.* 2010). Moreover, these patients also displayed an overgrowth of the facultative anaerobes, aerobes, and other unusual bacteria (Darfeuille-Michaud *et al.* 2004; Baumgart *et al.* 2007; Lupp *et al.* 2007). Thus, there is an increase in oxygen tension in the gut of IBD patients causing dysbiosis. This would favor the growth of oxygen-dependent bacteria like Corynebacteriaceae (CR conditions) and Lactobacillaceae (CZ conditions), which are present at high numbers in the *pirk;trabid* strain [for a review of the so-called “oxygen hypothesis” see Rigottier-Gois (2013)]. Therefore, constitutive immune activity in *Drosophila* causes a shift in the gut microbiota favoring aerobes, typical of an inflamed gut in humans.

Recent modeling and experimental analysis in zebrafish (Rolig *et al.* 2015) suggested that bacteria–bacteria interactions play the dominant role in determining community membership in gut microbiota, with no evidence for immune feedback on the bacterial populations. This is consistent with a model for self-regulating microbiota. However, our data indicate that this would not be the case in a pathologically inflamed intestine, where immunity imposes selection pressures. In this context, certain members of the microbiota would likely experience growth inhibition and other inflammation-adapted species would thrive. This is in line with previous observations in *Drosophila* (Ryu *et al.* 2008).

### Constitutively active immunity influences the microenvironment

Culturing *pirk;trbd* in the same microenvironment as *yw* influenced the diversity and the gut microbiota structure of the latter. Co-culture rapidly developed toward a mono-association with Acetobacteraceae. One conclusion from this data is that a fly strain with a constitutively active immune system was the one that had the major influence on the microenvironment and, by extension, on its nonsibling cohabitants. A second conclusion is that conditions of constitutively active immunity favor Acetobacteraceae in the gut microbiota. This bacterial family was mostly transferred by familial transmission in *yw*, *DGRP-208* and *dif-key* (compare Figure 1A with Figure 2A). In contrast, *pirk;trbd* flies had stably associated Acetobacteraceae even in CZ conditions.

Deciphering the mechanisms that shape and maintain microbial communities on mucosal surfaces may provide approaches to prevent or ameliorate a range of human diseases from diabetes and obesity to IBD. Our data show that constitutively active immunity can provoke changes in microbiota by influencing both the structure as well as the load of gut microbiota. In its turn, disrupted microbiota can influence gut physiology and life span. Age, genetics, microenvironment, and familial-transmitted bacteria are major factors of the above equilibrium. Based on the evolutionary conservation between fly and mammalian gut innate immunity, our results will help simplify and decode the influence of immune status and its interaction with the microenvironment in shaping the gut microbiota.

## Supplementary Material

Supplemental material is available online at www.genetics.org/lookup/suppl/doi:10.1534/genetics.116.190215/-/DC1.
