# Supplementary figures and images for: Interaction Between Familial Transmission and a Constitutively Active Immune System Shapes Gut Microbiota in Drosophila melanogaster
Source: Genetics. 2017 Apr 14;206(2):889–904. doi: 10.1534/genetics.116.190215 (PMC5499193; doi:10.1534/genetics.116.190215)

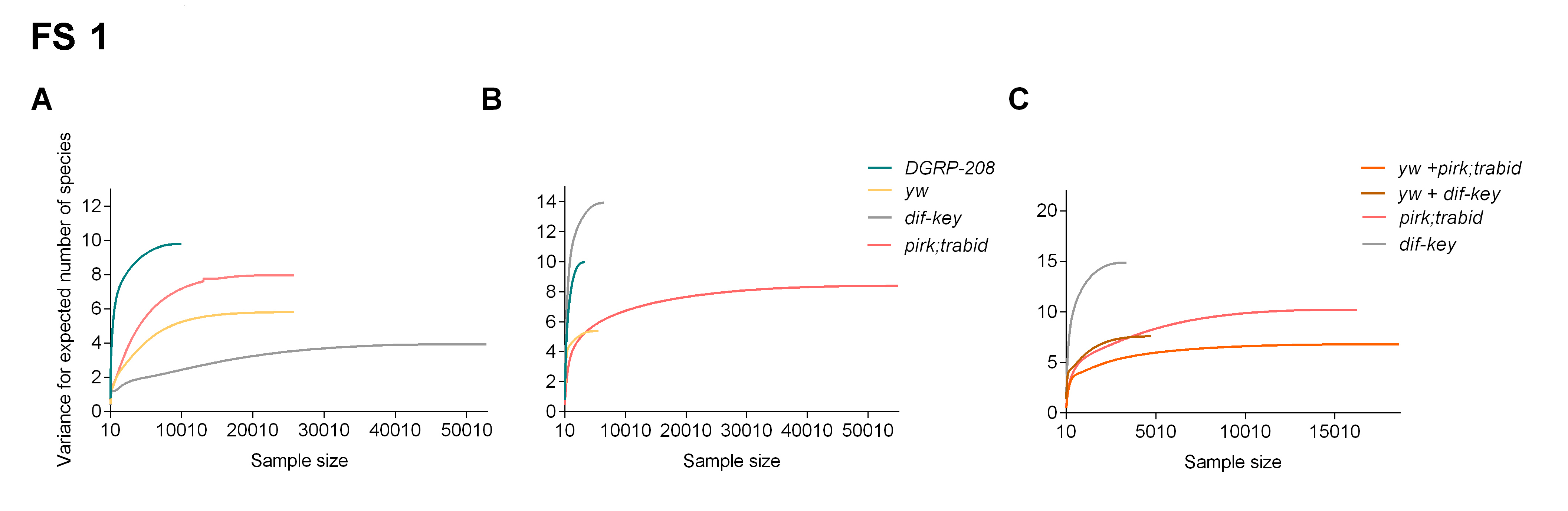

Supplement: Supplementary file 1 [file 889FigureS1.jpg]

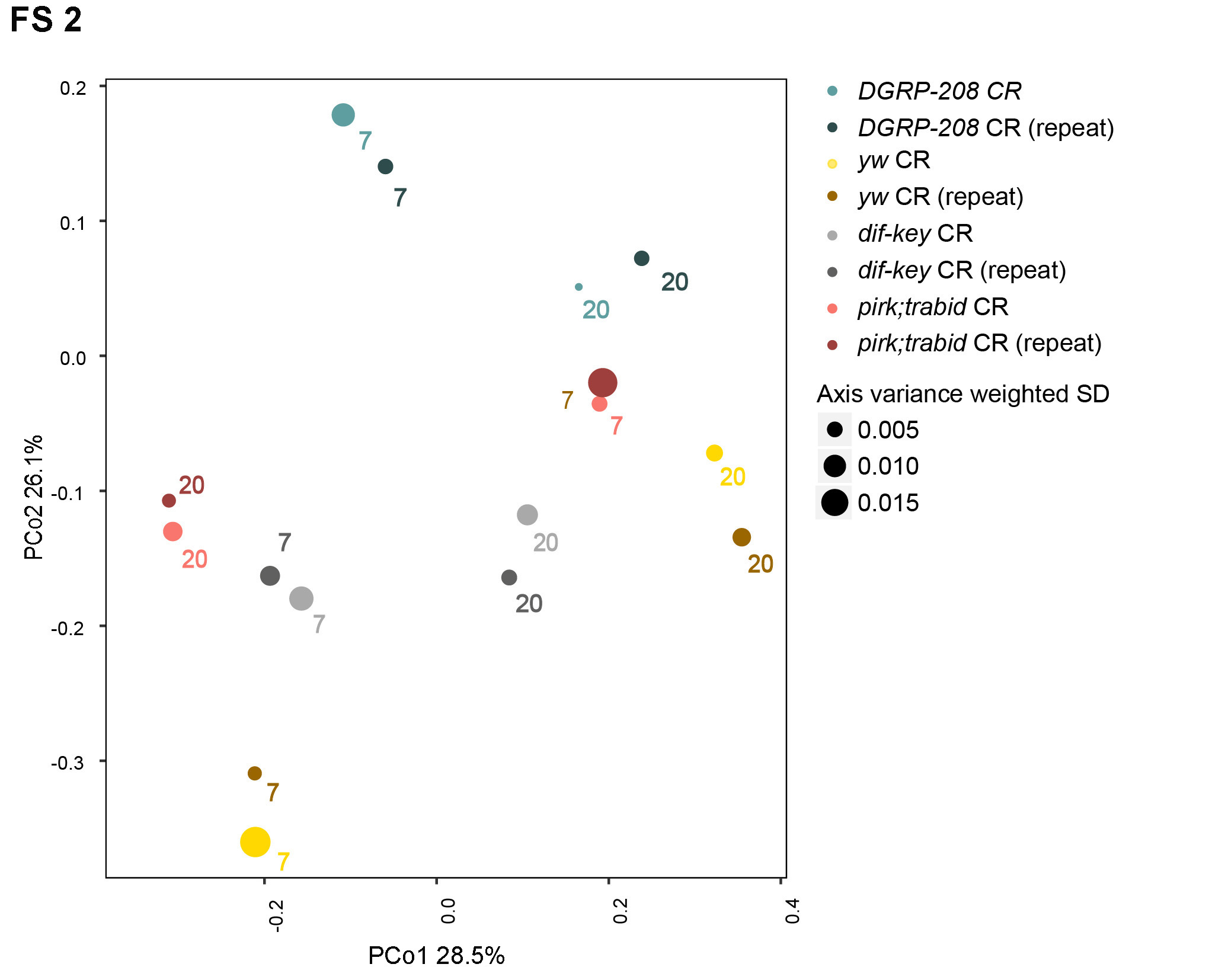

Supplement: Supplementary file 2 [file 889FigureS2.jpg]

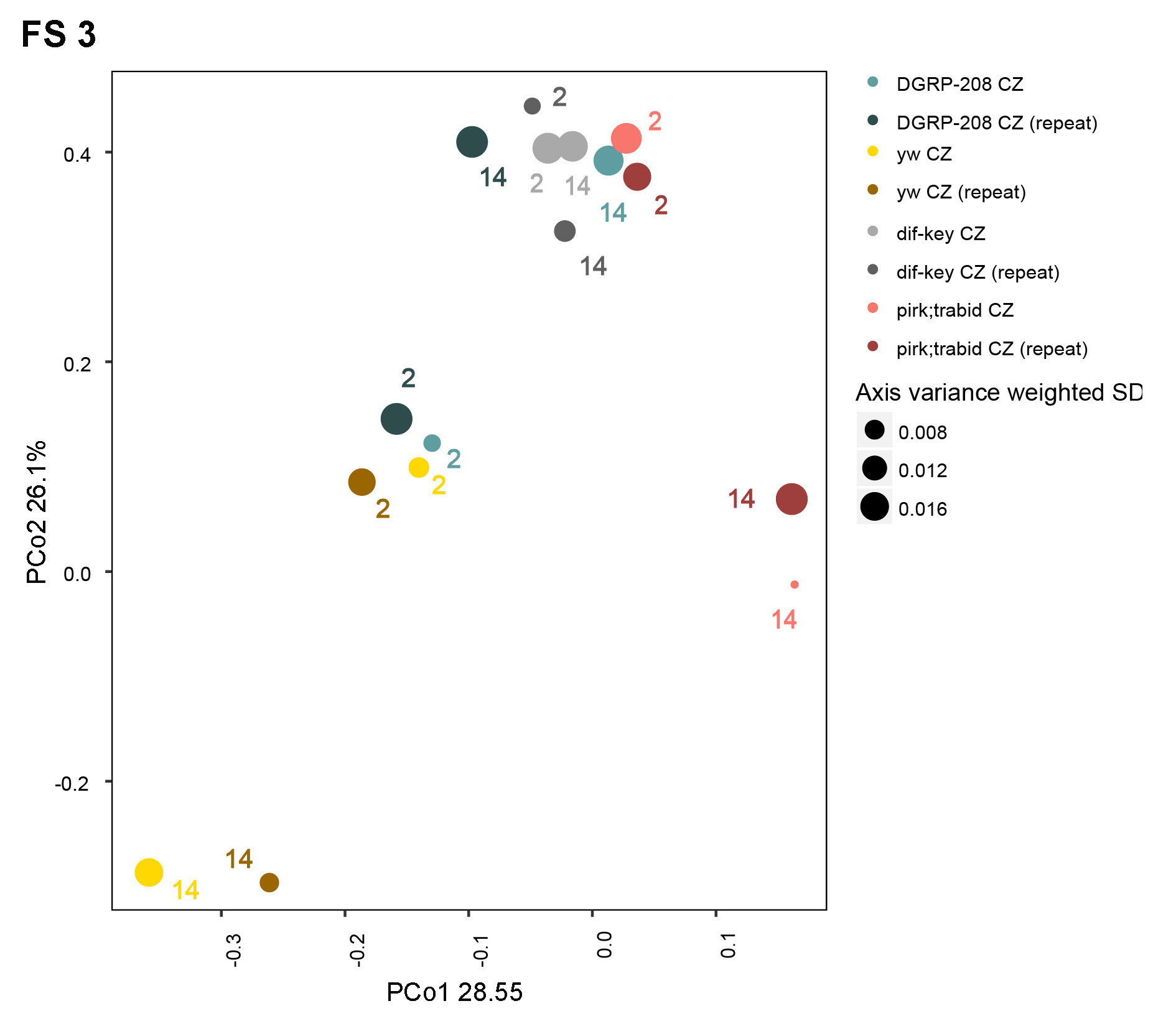

Supplement: Supplementary file 3 [file 889FigureS3.jpg]

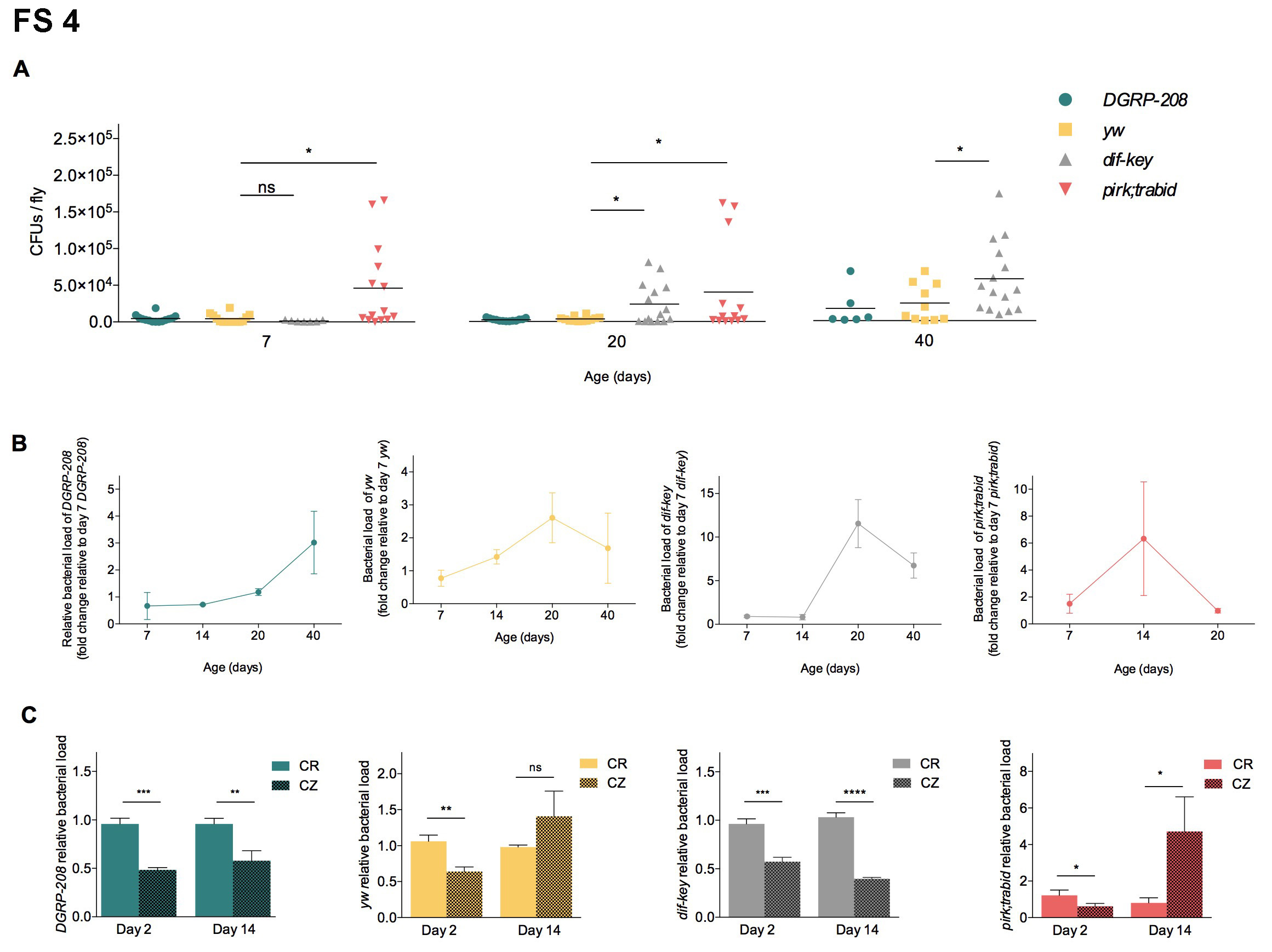

Supplement: Supplementary file 4 [file 889FigureS4.jpg]

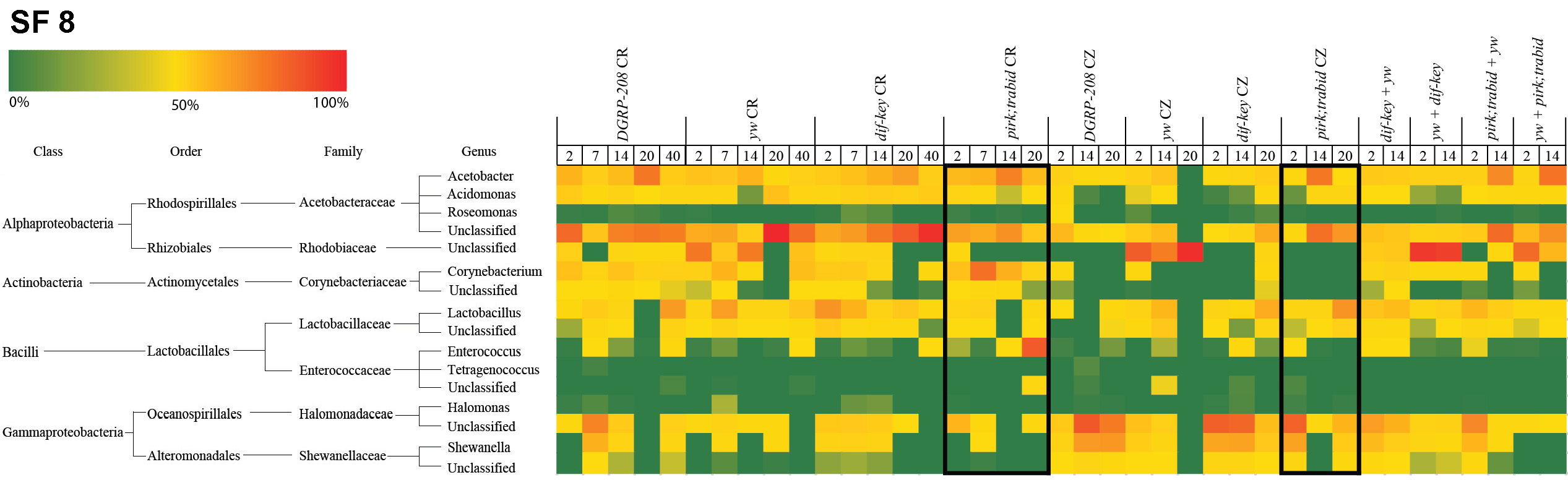

Supplement: Supplementary file 5 [file 889FigureS5.jpg]

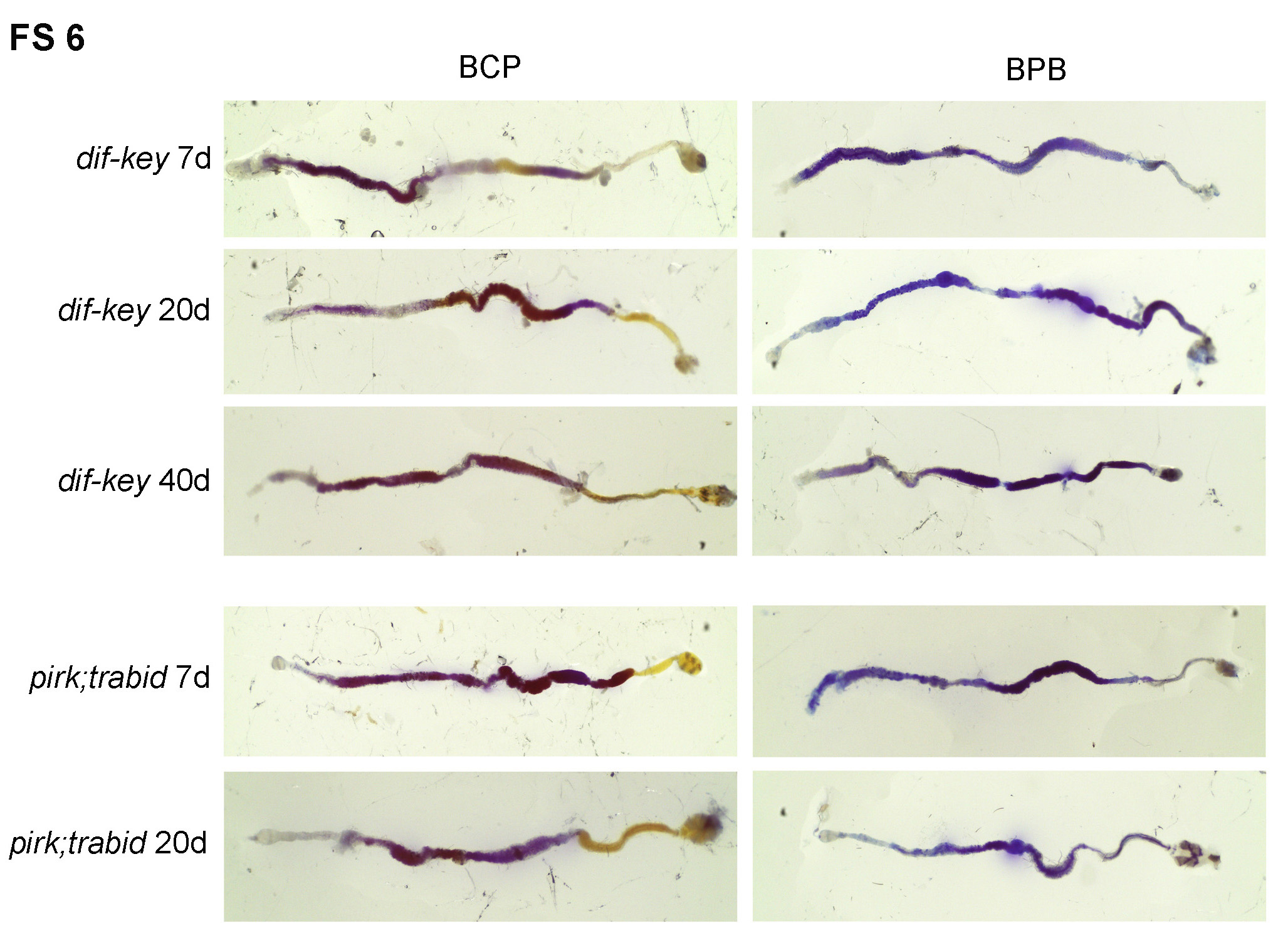

Supplement: Supplementary file 6 [file 889FigureS6.jpg]

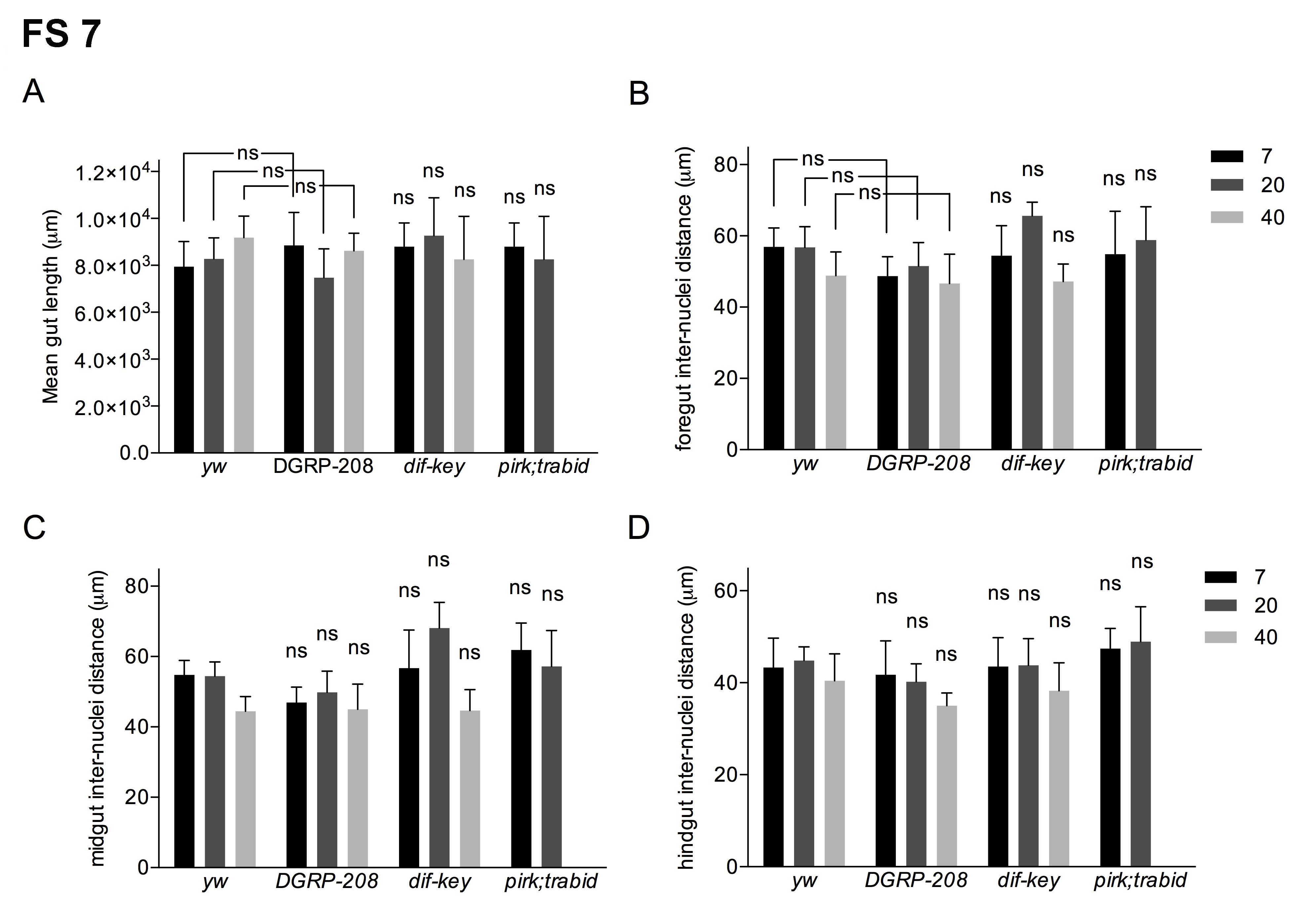

Supplement: Supplementary file 7 [file 889FigureS7.jpg]

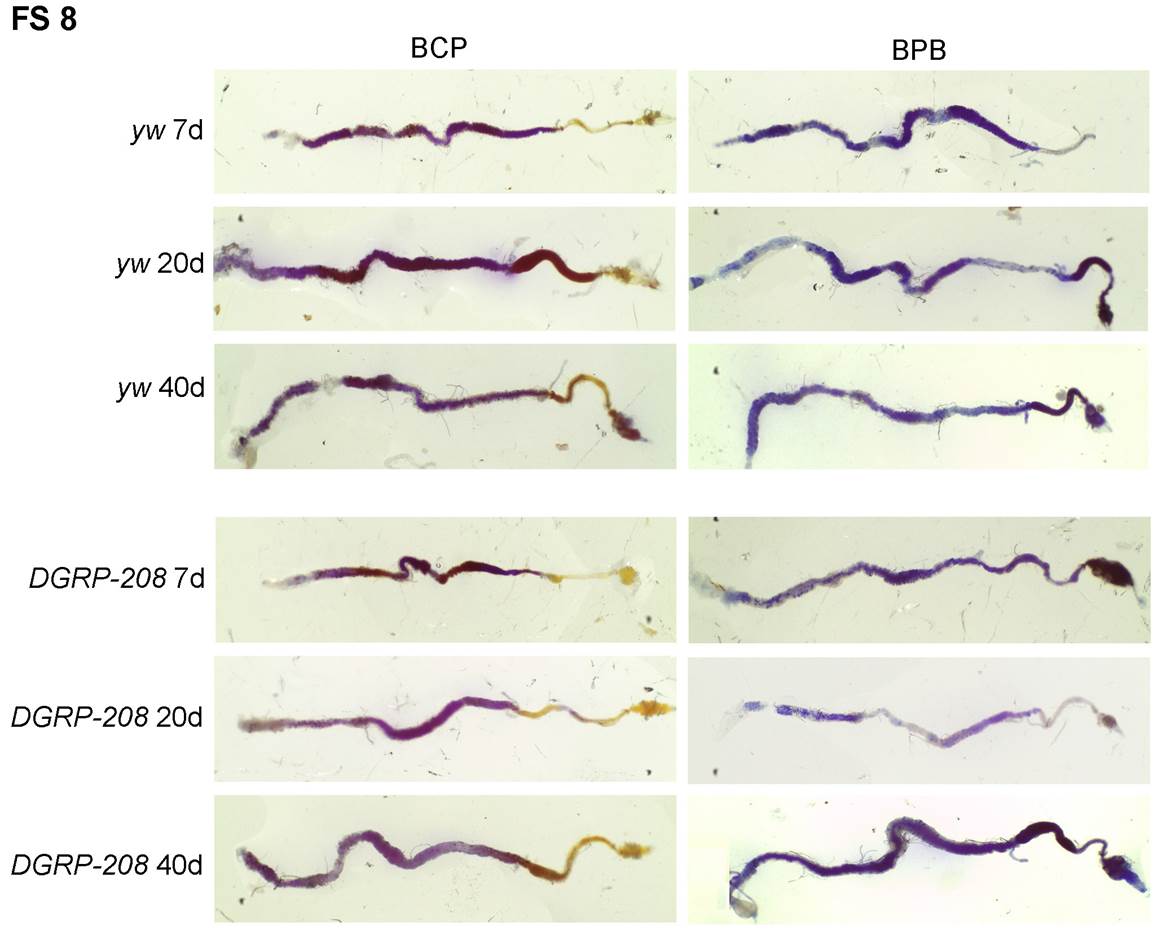

Supplement: Supplementary file 8 [file 889FigureS8.jpg]
