# Supplementary material for: Interaction Between Familial Transmission and a Constitutively Active Immune System Shapes Gut Microbiota in Drosophila melanogaster
Source: Genetics. 2017 Apr 14;206(2):889–904. doi: 10.1534/genetics.116.190215 (PMC5499193; doi:10.1534/genetics.116.190215)
Supplement: Supplementary file 9 [file 889TableS1.pdf]

Supplementary Table 1

Bacteria		DGRP-208		yw	
Family	Species	2-10 days	14-20 days	2-10 days	14-20 days
Acetobacteraceae	Acetobacter aceti			+	+
	Acetobacter pasteurianus	+	+	+	+
Lactobacillaceae	Lactobacillus brevis			+	+
	Lactobacillus pentosus	+		+	
	Lactobacillus plantarum	+	+	+	+
Micrococcaceae	Micrococcus luteus	+			

Bacteria		dif-key		pirk-trabid	
Family	Species	2-10 day	14-20 day	2-10 day	14-20 day
Acetobacteraceae	Acetobacter aceti			+	+
	Acetobacter pasteurianus	+	+	+	+
Lactobacillaceae	Lactobacillus brevis	+		+	
	Lactobacillus plantarum	+	+	+	
Micrococcaceae	Micrococcus luteus			+	
Moraxellaceae	Moraxella osloensis			+	
Paenibacillaceae	Paenibacillus taichungensis	+			
Comamonadaceae	Variovorax paradoxus	+			
