# Supplementary material for: Interaction Between Familial Transmission and a Constitutively Active Immune System Shapes Gut Microbiota in Drosophila melanogaster
Source: Genetics. 2017 Apr 14;206(2):889–904. doi: 10.1534/genetics.116.190215 (PMC5499193; doi:10.1534/genetics.116.190215)
Supplement: Supplementary file 10 [file 889TableS2.pdf]

Supplementary Table 2

Bacteria		*DGRP-208*		*yw*	
Family	Species	2-10 days	14-20 days	2-10 days	14-20 days
Acetobacteraceae	*Acetobacter aceti*			+	+
	Acetobacter pasteurianus	+	+	+	+
Lactobacillaceae	*Lactobacillus brevis*			+	+
	Lactobacillus pentosus	+		+	
	Lactobacillus plantarum	+	+	+	+
Micrococcaceae	*Micrococcus luteus*	+			

Bacteria		*dif-key*		*pirk-trabid*	
Family	Species	2-10 day	14-20 day	2-10 day	14-20 day
Acetobacteraceae	*Acetobacter aceti*			+	+
	Acetobacter pasteurianus	+	+	+	+
Lactobacillaceae	*Lactobacillus brevis*	+		+	
	Lactobacillus plantarum	+	+	+	
Micrococcaceae	*Micrococcus luteus*			+	
Moraxellaceae	*Moraxella osloensis*			+	
Paenibacillaceae	*Paenibacillus taichungensis*	+			
Comamonadaceae	*Variovorax paradoxus*	+			
